# Quantitative interactome analysis reveals a chemoresistant edgotype

**DOI:** 10.1038/ncomms8928

**Published:** 2015-08-03

**Authors:** Juan D. Chavez, Devin K. Schweppe, Jimmy K. Eng, Chunxiang Zheng, Alex Taipale, Yiyi Zhang, Kohji Takara, James E. Bruce

**Affiliations:** 1 Department of Genome Sciences, University of Washington, Seattle, Washington 98195, USA; 2 Department of Pharmaceutical Sciences, Himeji Dokkoyo University, Himeji, Hyogo 670-8524, Japan

## Abstract

Chemoresistance is a common mode of therapy failure for many cancers. Tumours develop resistance to chemotherapeutics through a variety of mechanisms, with proteins serving pivotal roles. Changes in protein conformations and interactions affect the cellular response to environmental conditions contributing to the development of new phenotypes. The ability to understand how protein interaction networks adapt to yield new function or alter phenotype is limited by the inability to determine structural and protein interaction changes on a proteomic scale. Here, chemical crosslinking and mass spectrometry were employed to quantify changes in protein structures and interactions in multidrug-resistant human carcinoma cells. Quantitative analysis of the largest crosslinking-derived, protein interaction network comprising 1,391 crosslinked peptides allows for ‘edgotype' analysis in a cell model of chemoresistance. We detect consistent changes to protein interactions and structures, including those involving cytokeratins, topoisomerase-2-alpha, and post-translationally modified histones, which correlate with a chemoresistant phenotype.

## Abstract (web summary)

Changes in protein–protein interactions result in changes to cellular phenotype. Here the authors use crosslinking mass spectrometry to derive a quantitative protein interaction network in drug-sensitive and -resistant HeLa cells, and uncover a chemoresistant ‘edgotype'.

## Graphical abstract

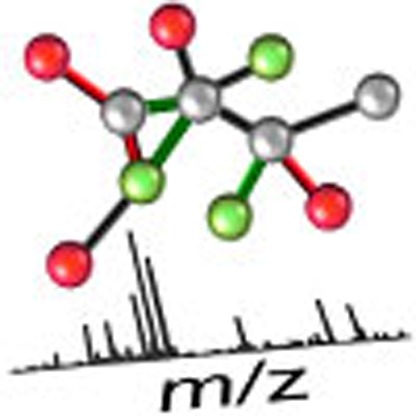

Chemotherapy, along with radiotherapy and surgery, is one of the principal treatments for cancer patients. During treatment, matching chemotherapeutic agents with susceptible tumours is critical to clinical efficacy^1^. Modern large-scale measurements based on genomics and proteomics technologies have significantly increased the ability to identify novel genes and signalling networks that are involved in the responsiveness of tumours to particular chemotherapeutic agents. However, intrinsic and acquired resistance to chemotherapy limits the effectiveness of treatment. Tumours or cells that initially were responsive to therapy can acquire resistance due to mutations that can occur during chemotherapy, adaptive responses to chemotherapy, or chemotherapy-induced selection of a resistant minor subpopulation of cells present in the original heterogeneous tumour. Therefore, chemoresistance represents a significant barrier to improved long-term outcome for many cancer patients.

A variety of mechanisms contribute to multidrug-resistant (MDR) phenotypes including: decreased drug uptake, increased drug efflux, activation of detoxifying systems, activation of DNA repair mechanisms and evasion of drug-induced apoptosis^2^. Here we chose to study a MDR HeLa cell line (HeLa/SN100) with demonstrated resistance to 16 different chemotherapeutic agents which was developed by exposure to 100 nM of SN-38, the active metabolite of irinotecan^3^. Irinotecan is a derivative of camptothecin and is widely used for the treatment of colorectal cancer, ovarian and small cell lung carcinoma. Irinotecan is converted by carboxylesterases into the active form SN-38, which exerts its cytotoxic activity through inhibition of topoisomerase 1 (TOP1) religation activity and indirectly results in DNA double strand breaks (DSBs)^4^. SN-38 resistance has been shown to result from drug efflux^5^,^6^, reduced TOP1 expression^7^, TOP1 mutations^8^,^9^, suppression of apoptotic pathways^10^ and activation of survival pathways^11^. Thus, mechanisms relevant to SN-38 resistance are complex and likely to involve conformational and interaction changes among many proteins.

The human proteome has been estimated to comprise ∼130,000 protein–protein interactions (PPIs) at any given time^12^. Through these interactions, cells are able to carry out a vast array of functions and adapt to environmental conditions. Yet, the majority of these interactions have not been mapped and the proteins involved lack molecular structural information necessary for their characterization. Mapping of PPI networks, or the ‘interactome'^13^ is a goal with promise to improve our understanding of the molecular mechanisms of disease and chemoresistance. Improved comprehension of protein interaction networks to help understand functional phenotypes requires new capabilities that enable visualization of changes at the protein interaction network level. In an interactome network model consisting of nodes and edges, quantification of interactions (edges) can provide an ‘edgotype' for the MDR phenotype^14^.

Chemical crosslinking with mass spectrometry is a technique that can be used to identify interacting proteins. The formation of new covalent bonds between reactive amino acid side chains on the surfaces of proteins, stabilizes protein structures and provides information on the architecture of protein complexes^15^,^16^,^17^. Previous efforts have demonstrated the utility of protein interaction reporter (PIR)-crosslinking technology to construct interactome network maps in complex biological systems such as intact virions^18^, *Escherichia coli*^19^,^20^ and human cells^21^. The edges in these networks represent proximal amino acid residues containing both individual protein conformational information for intra-protein linkages, as well as protein interaction and protein complex structural information for inter-protein linkages. Comparative crosslinking experiments on purified proteins or protein complexes have demonstrated the potential of the technique to quantify ligand binding^22^ or conformational changes induced by post-translational modifications (PTMs)^23^. Several possibilities exist to explain differences in the relative abundance of crosslinked peptide pairs including: protein conformational rearrangement, changes in levels of complex formation between interacting proteins, or PTMs at crosslinked lysine residues precluding reaction with the crosslinker. To enable edgotype studies of the MDR phenotype, we applied PIR technology^24^,^25^ and stable isotope labelling by amino acids in cell culture (SILAC)^26^ to compare sensitive and chemoresistant cancer cells. The approach presented here builds on earlier work and enables quantitative measurements of interactions and structures in cells by incorporation of stable isotopic labelling. Quantification of crosslinked peptides enables measurement of an edgotype map^14^ in which protein conformational changes and interaction level changes can now be observed between biological phenotypes on a large scale.

Herein, we demonstrate consistent quantification of crosslinked peptides across biological samples enabling assembly of a quantitative interaction network. Edgetic network analysis reveals changes to protein interactions and structures that correlate with a chemoresistant phenotype. These include epigenetic mark-induced structural changes to histone H3, increased interactions between the intermediate filament (IF) components keratins 8/18, and changes to DNA topoisomerase-2-alpha that correlate with increased enzymatic activity. Collectively, we show that quantitative crosslinking with mass spectrometry provides unique insight into the state of the proteome and serves as a new tool for characterizing biological phenotypes.

## Results

### Quantification of crosslinked peptides

**Figure 1:**
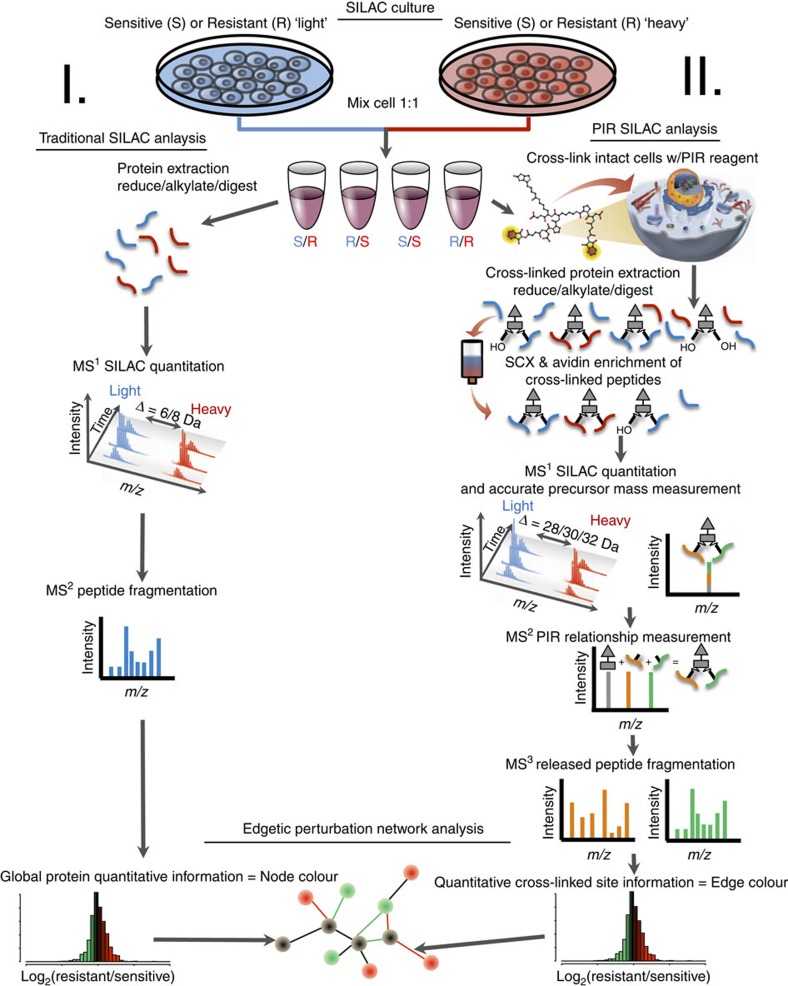
Quantitative *in vivo* crosslinking experimental flow chart. Drug-sensitive HeLa (S) and drug-resistant HeLa/SN100 (R) cells were cultured in isotopically light and/or heavy stable isotope labeling by amino acids in cell culture (SILAC) media. Light and heavy cells were mixed at 1:1 ratio and either subjected to **I**. Traditional quantitative SILAC analysis to obtain relative global protein abundances or **II**. *In vivo* crosslinking with PIR crosslinker. Crosslinked proteins were extracted reduced, alkylated and digested. Crosslinked peptides were purified by a combination of strong cation exchange (SCX) and avidin affinity chromatography and analysed using ReACT^10^. Data from the traditional SILAC and crosslinking SILAC experiments were merged into a quantitative interaction network providing an edgotype for multidrug resistance in HeLa cells.

In this study comparative SILAC-based quantitative proteomics analysis was performed on drug-sensitive and -resistant HeLa cells using both non-crosslinked, tryptic digest samples, as well as enriched PIR-crosslinked peptide samples. The general experimental strategy is outlined in Fig. 1. Our two pronged approach provides both relative quantitative information on global protein abundance levels and relative quantitative information on protein conformational and interaction level changes. When quantifying crosslinked peptides, the direction and magnitude of observed abundance change may vary from protein expression levels measured by traditional SILAC. These differences reveal information on proteome changes that are unobservable by traditional quantitative measurements alone. With a few exceptions, crosslinking studies carried out to date have been purely qualitative in nature. We previously used label-free mass spectrometry (MS^1^)-based quantitation to monitor binding levels of two competitive peptide ligands with immobilized calmodulin^22^. Schmidt and Robinson^23^ used an isotopically labelled crosslinker (BS3-do/d4) to quantify conformational changes on ATP synthase isolated from spinach chloroplasts with various levels of phosphorylation. These studies demonstrated that quantifying crosslinked peptides can provide unique and valuable information on the conformational and complex state of protein systems.

### Identification of *in vivo* crosslinked proteins

In total, 1,391 unique crosslinked peptide pairs, consisting of 1,461 crosslinked sites from 437 proteins were identified in these efforts (Supplementary Data 1), making this the largest crosslinking data set from mammalian cells to date. The sequences for crosslinked peptide pairs were identified by searching the mass spectrometric data against a stage 1 database (Supplementary Data 2 and Supplementary Methods) consisting of 3,348 putative PIR-reactive proteins and mapping the sequences back to PIR mass relationships identified during liquid chromatography–MS (LC–MS) data acquisition^19^,^21^,^27^. The false discovery rate (FDR) for these 1,391 identified crosslinked peptide pairs is estimated to be ≤1% using a target/decoy search strategy (see Supplementary Methods for details)^27^. Although greatly expanded in scope, a high degree of overlap was observed between the crosslinked proteins identified in this study and those from previous *in vivo* crosslinking studies, including 265 crosslinked peptide pairs from HeLa cells^21^, and 240 crosslinked peptide pairs from HEK293 cells^28^. This encouraging observation, illustrates the robustness and reproducibility of the *in vivo* crosslinking approach. Twenty-five percent (354) of the 1,391 crosslinked peptide pairs correspond to intermolecular (inter-protein and homooligomer) interactions, while the remaining 75% (1037) are intra-molecular (intra-protein) interactions. These numbers are highly consistent with previous studies conducted using HeLa^21^, *E.coli*^19^ and *Pseudomonas aeruginosa*^27^ cells. The 354 intermolecular crosslinked peptide pairs represent 189 unique binary protein interactions, 144 of which are inter-protein interactions, while 45 are homooligomer interactions. Eighty seven of the 354 intermolecular crosslinked peptide pairs are unambiguous homodimers, defined as two crosslinked peptides that share overlapping sequence only occurring once in a given protein sequence, thereby requiring a minimum of two protein subunits. These results highlight the fact that chemical crosslinking is a powerful approach for definitively identifying protein homooligomer interactions, which are often difficult to characterize by other techniques. Fifty-one binary protein interactions are supported by two or more crosslinked peptide pairs, while the remaining 138 PPIs are represented by a single crosslinked peptide pair. One challenge with identifying crosslinked peptides is assigning peptide sequences to proteins due to the fact that protein assignment often relies on a minimal set of peptides. In the current study 86% (1253) of the 1,452 peptides identified were unique to a single protein in our protein database. This is partially due to the fact that every crosslinked peptide will contain at a minimum one internal lysine (missed tryptic cleavage site), leading to longer peptide sequences that are more likely to be unique to a specific protein. Of the remaining 14% (198/1,452) of peptide sequences that occur in more than one protein sequence, 106 were shared across just two isoforms, 82 were shared between 3 and 5 isoforms, while the remaining 10 were comprised of highly redundant (shared by >5 proteins) peptide sequences or short peptides (<5 residues) that lack specificity. The full list of peptide redundancy is included in Supplementary Data 1. The crosslinked proteins identified represent all major subcellular compartments with the majority being from the nucleus, membrane and cytosol with smaller numbers from other compartments agreeing well with our previously published results (Supplementary Fig. 1)^21^. These data were uploaded into XlinkDB^29^ (http://brucelab.gs.washington.edu/xlinkdb) where Euclidian distance between lysine alpha carbons was mapped onto existing structures in the Protein Data Bank (PDB). A total of 357 crosslinked peptide pairs were mapped to structures with a median Euclidean distance of 16 Å (Supplementary Fig. 2). The remaining 1,034 crosslinks exist in proteins or regions of proteins for which there was no structural data in the PDB. XlinkDB also compared the list of interactors to known PPIs from the following databases; MIPS, DIP, IntAct, MINT, HPRD and BioGRID. A distribution of nodal distance is included in Supplementary Fig. 3. While the largest fraction of identified crosslinks were assigned as intra-protein interactions, the majority of inter-protein interactions were previously known to be direct interactors (nodal distance=0) or share a single common interacting partner (nodal distance=1) in at least one of the aforementioned databases.

### Quantitative interaction network analysis

**Figure 2:**
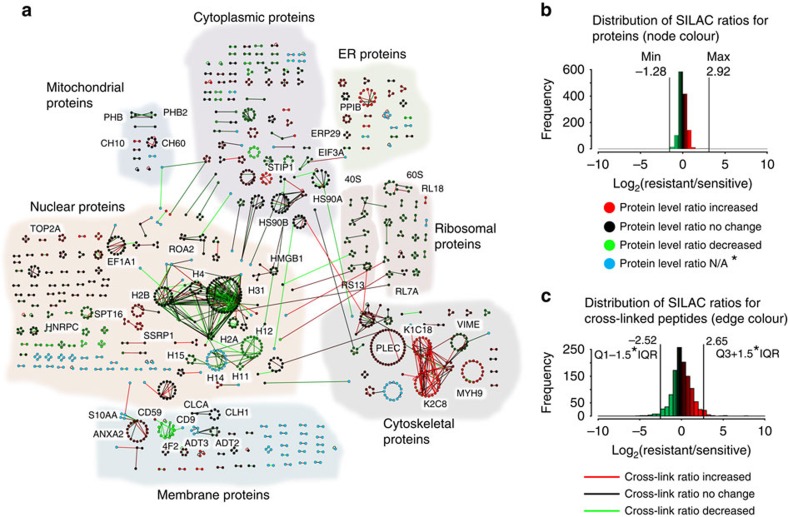
Quantitative interaction network. (**a**) Quantitative crosslinked peptide interaction network consisting of 1,308 nodes representing unique crosslinked peptides connected by 1,166 edges representing crosslinks between two peptides. Loop edges connecting the same node to itself represent unambiguous homodimer crosslinked peptide pairs. Nodes are grouped in circular clusters according to their protein assignment. A force-directed layout reveals clusters of linked proteins corresponding to general functional class or subcellular localization, which are indicated by coloured shapes encompassing clusters of linked proteins. (**b**) Distribution of log_2_(Resistant/Sensitive; R/S) ratios (average values obtained from analysis of six biological replicates) obtained by traditional SILAC analysis representing relative global protein level changes between the HeLa and HeLa/SN100 cell lines. The histogram is colour coded to match the node colour in the network. Red coloured nodes represent proteins with increased relative expression levels in the chemoresistant cell line versus the sensitive cell line, while green coloured nodes represent proteins with decreased relative expression levels in the chemoresistant cell line versus the sensitive cell line. Black coloured nodes represent proteins that did not have a measureable change between the two cell lines. *Blue nodes represent proteins for which no R/S ratio was obtained. (**c**) Distribution of log_2_(R/S) ratios (average values obtained from analysis of five biological replicates) for the crosslinked peptide pairs quantified between the HeLa and HeLa/SN100 cell lines, colour coded according to match the edge colour in the network. Red, green and black coloured edges correspond to relative changes in crosslinked peptide levels between the resistant and sensitive cell lines with the same colour coding scheme as for the nodes.

**Figure 3:**
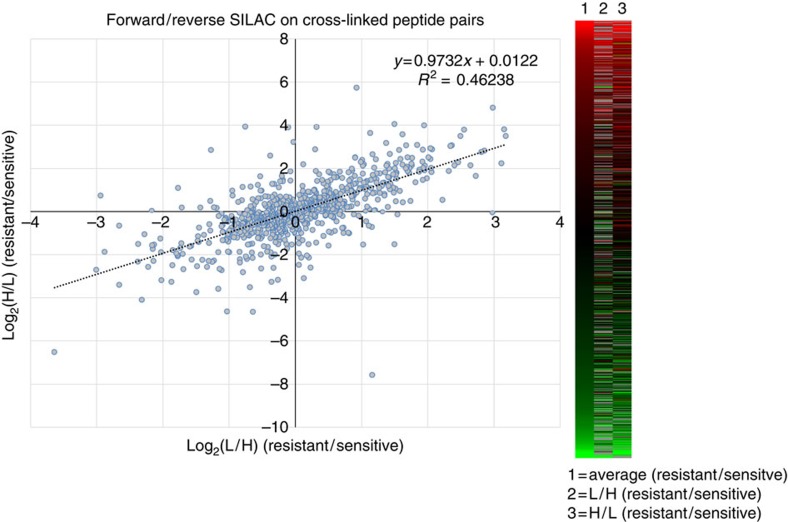
Quantitative reproducibility of crosslinked peptides. Scatterplot of log_2_(light/heavy) versus log_2_(heavy/light) values for *n*=767 crosslinked peptide pairs out of 1,166 total (66%). Those that were only observed in forward or reverse SILAC experiments were omitted from the scatterplot but can be seen as grey entries in the heatmap.

The novel combination of PIR technology and SILAC allowed for quantification across the protein interaction network for both the edges (mean crosslinked peptide pair log_2_(R/S) ratio) and nodes (mean log_2_(R/S) protein ratio from traditional SILAC analysis). SILAC MS^1^-based quantification was carried out for 1,166 of these crosslinked peptide pairs between chemoresistant and sensitive cells, while the remaining 225 crosslinks were only identified in control samples consisting of a 1:1 mixture of light- and heavy-drug-sensitive cells. This quantitative interaction network consists of 1,308 nodes representing unique peptide sequences, connected by 1,166 edges representing the crosslinks between the peptides (Fig. 2a). Peptide nodes were grouped into circular clusters according to their corresponding protein, with 374 proteins represented in this network. Log_2_(R/S) ratios for the crosslinked peptides varied from −5.15 to +7.58 with a s.d. of 1.25 (Fig. 2b,c and Supplementary Data 1). Protein ratios spanned a log_2_(R/S) range from −5.55 to 4.61 with a s.d. of 0.7. SILAC ratios were obtained for 76% (285/374) of the crosslinked proteins in the network shown in Fig. 2. Normal distributions were observed for the SILAC ratios for both the crosslinked peptides (Fig. 2b) and protein ratios (Fig. 2c), indicating that the majority of crosslinked peptides and individual protein levels were unaltered between the drug-resistant and -sensitive cells. The consistency of crosslinked peptide levels observed in the drug-sensitive and -resistant cell lines was used to identify changes in interactions with defined confidence intervals. To ensure reproducibility of the crosslinking SILAC approach, samples were analysed in five biological replicates, including isotope label swapping. The values obtained after swapping the direction of the heavy isotope amino acid labelling, that is, (HeLa/SN100(light))/(HeLa(heavy)) versus (HeLa(light))/(HeLa/SN100(heavy)), were compared to determine the consistency of crosslinked peptide quantitation in biological replicates (Fig. 3). General agreement was observed between the forward and reverse PIR SILAC experiments with a linear regression slope of 0.97, and an *R*^2^ value of 0.46.

To gauge the variability in the achieved quantitative measurements, confidence intervals (*α*=0.05, CI) were calculated for the log_2_(R/S) SILAC ratios obtained for crosslinked peptide pairs and protein levels. The variability of the PIR SILAC quantitative measurements were higher for crosslinked peptides (median CI=0.82) than for traditional SILAC measurements on tryptic peptides (median CI=0.14; Supplementary Fig. 4). This could be due to a number of factors specific to the crosslinking experiment including changes to: protein conformations, PTM occupancy levels and protein accessibility and/or reactivity with the crosslinker due to changes in subcellular location or physicochemical environment. Nonetheless given the large number of variables and steps in sample preparation involved, the data show good agreement between *in vivo* crosslinking in cells across biological replicates (Fig. 3 and Supplementary Fig. 4). Crosslinked peptide pairs which change significantly between the chemoresistant and sensitive cells, were defined as those that had an absolute log_2_(R/S) value >1 and a CI <1 (Supplementary Fig. 4).

Importantly, these results demonstrate for the first time that *in vivo* crosslinking can achieve a level of consistency necessary for quantitation of large numbers of crosslinked peptide pairs across biological samples. If crosslinking on cells yielded random links between proteins that did not interact with high frequency and specific orientation to allow the identified linkages to be formed, one would expect that multiple biological replicates would yield sets of crosslinked peptide pairs with little to no agreement across replicates. The presented results show that this is not the case since crosslinked peptide pairs were repeatedly observed in at least four sets of crosslinked cultured cells (two sensitive and two resistant; Fig. 3). Furthermore, the intensity ratios between replicate resistant/sensitive comparisons show a high degree of consistency and indicate that statistical measures can be applied to *in vivo* crosslinked peptide pair ratios to identify significant changes. Therefore, this approach was used to reveal novel large-scale changes in protein structures and interactions in cells.

### Edgotype analysis reveals changes to DNA repair machinery

Proteins involved in DNA binding and repair pathways have been implicated in the mechanism of resistance to topoisomerase inhibitors^30^. Changes in histone conformations, interactions and PTMs are important factors in localizing proteins involved in DNA damage response to sites of broken DNA within the chromatin structure^31^. Therefore, altered levels of crosslinked peptides in histones, DNA-binding proteins and repair enzymes might be anticipated from *in vivo* crosslinking comparative analysis of cells that are sensitive and resistant to topoisomerase inhibitors.

**Figure 4:**
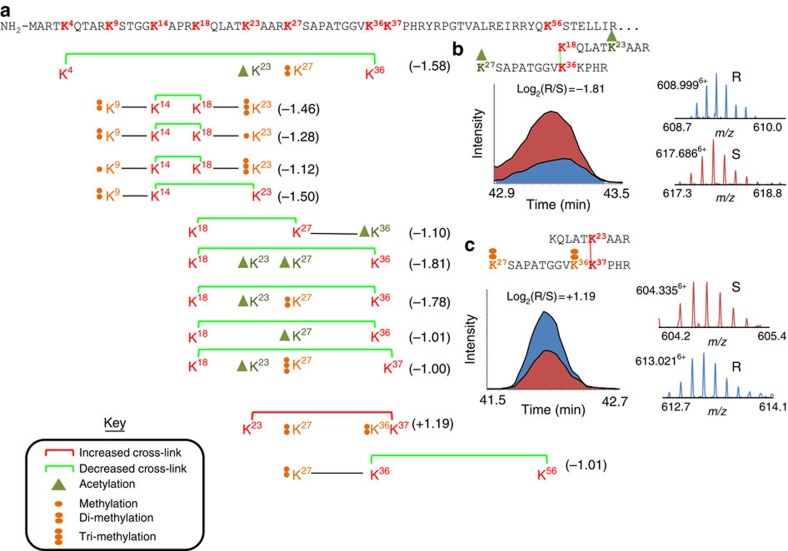
Quantitation of histone H3 crosslinked peptides with PTMs. (**a**) Map of intra-protein crosslinked peptide pairs from histone H3 (H31_Human) that were quantified with a Log_2_(Resistant/Sensitive) magnitude >1 with a 95% CI <1. Crosslinked residues are indicated by red bold. Residue numbering is according to canonical histone PTM nomenclature (UniProt-1)^38^ (**b**) Extracted ion chromatograms for the isotopically light and heavy crosslinked peptide pair linking K18 with K36 and containing acetylation modifications on K23 and K27. (**c**) Extracted ion chromatograms for the isotopically light and heavy crosslinked peptide pair linking K23 with K37 and containing dimethyl modifications on K27 and K36.

Histone H3 was the most heavily crosslinked protein observed in this study with a total of 153 unique crosslinked peptide pairs including 43 intra-protein linkages (Supplementary Data 1). The ability to detect PIR-crosslinked peptides containing PTMs on histones was previous demonstrated^21^. Here, 30 of the identified 43 intra-crosslinked peptides from histone H3 contained one or more PTMs including mono-, di- or tri-methylation on lysine, acetylation of lysine, or dimethyl arginine on one of seven unique sites of H3. These efforts add a quantitative dimension to these measurements, providing evidence for PTM-modulated conformational changes in histones. Histone modifications are important regulators of gene expression and chromatin remodelling and there is evidence that they play a role in chemoresistance in cancer^32^,^33^. Furthermore, genome-wide sequencing efforts have identified mutations at two specific sites in histone H3 (K27M and G34R/G34V) that promote development of high-grade paediatric gliomas which are highly resistant to treatment and have poor prognosis^34^, underpinning the importance of the molecular structure of the H3 N-terminal tail. Drugs that modulate PTMs on histones, including histone deacetylase (HDAC) inhibitors and lysine demethylase inhibitors, have shown promise in overcoming chemoresistance in certain cases^35^,^36^,^37^. Global levels of histone H3 did not significantly change between the chemoresistant and sensitive cells with a log_2_(R/S) of −0.10. However, several crosslinked histone H3 peptides were observed to have altered levels between the chemoresistant and sensitive cells. The crosslinked peptides from histone H3 that displayed altered levels with chemoresistance and were observed to carry specific PTMs are illustrated in Fig. 4. All of these crosslinked sites were located in the highly disordered N-terminal tail of histone H3 for which no crystal structure is available. PIR crosslinking and SILAC data revealed H3-tail conformational shifts caused by various PTMs that are differentially regulated in the chemoresistant phenotype. For example, the crosslink between K19 and K37 including the PTMs (H3K23Ac and H3K27Ac) was decreased in the MDR cells, log_2_(R/S)=−1.81, while the crosslink between K23 and K37 including dimethylation modifications at K27 and K36 increased with chemoresitance (log_2_(R/S)=1.19; Fig. 4b,c). Note that the amino acid residue numbering scheme from the UniProt database (initial Met=1) is used for all cases except for histone proteins which are numbered according to the canonical histone mark nomenclature (UniProt—1)^38^. Interestingly, the K23–K37 linkage was the only PTM containing intra-crosslinked H3 peptide pair with increased levels in the chemoresistant cell line. Dimethylation of K27 (H3K27me2) was identified as one of the prominent histone marks of transcriptional repression^39^. Dimethylation of K36 (H3K36me2) was observed at the site of DNA DSBs and helps in the non-homologous end joining (NHEJ) repair process^40^. Differential modification of H3K36 has been described as a molecular switch where methylation is proposed to promote a closed chromatin structure and facilitate NHEJ, while acetylation promotes an open chromatin structure, reducing binding of DSB repair machinery^41^. The proposal made by Pai *et al*., is consistent with our observation of decreased levels of the crosslink between K18 and K27 containing H3K36ac (Fig. 4).

**Figure 5:**
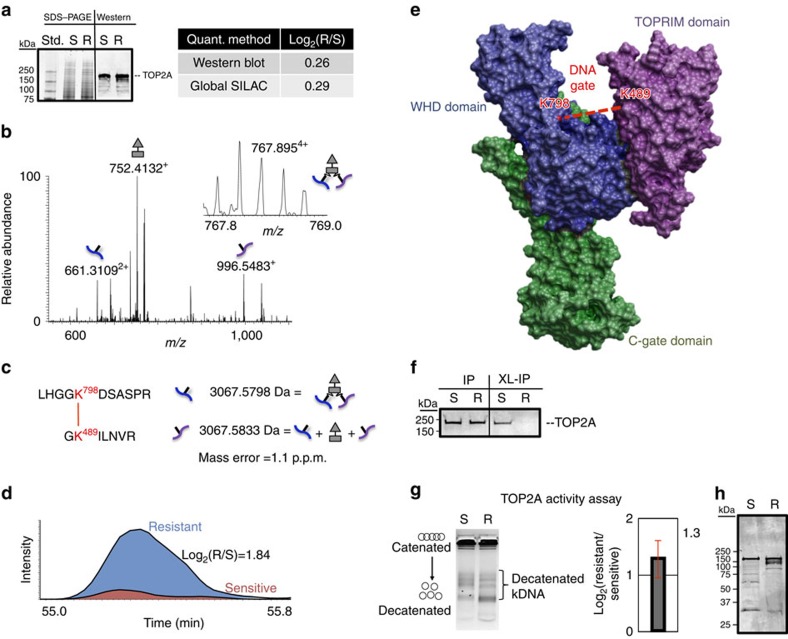
Correlation between DNA topoisomerase A2 crosslink and activity levels. (**a**) Coomassie stained one-dimensional SDS–PAGE of drug-sensitive (S) and -resistant (R) full cell lysates and western blot for TOP2A. Quantitative measurements for TOP2A obtained by western blot (*n*=6) and global SILAC (*n*=6) are in excellent agreement and indicate similar expression levels of TOP2A in the sensitive and resistant cells. (**b**) High-resolution MS^2^ spectrum indicating the *m*/*z* values for the released peptide ions and the reporter ion with MS^1^ insert illustrating the 4+ precursor ion. (**c**) PIR mass relationship indicating the high mass accuracy measurement of the precursor ion and released peptides in A with a mass error of 1.1 p.p.m. (**d**) Extracted ion chromatograms for the MS^1^ signal from the precursor ions from the resistant cells (*m*/*z* 767.89495) and sensitive cells (*m*/*z* 774.912). Increased levels (log_2_(Resistant/Sensitive; R/S)=1.84) of this crosslinked peptide pair were measured in the MDR cell line. (**e**) The crosslink observed between K489 and K789 spans the DNA-binding gate mapped onto PDB structure 4FM9. (**f**) Western blot analysis of the immunoprecipitation of TOP2A with and without crosslinking from drug-sensitive (S) and -resistant (R) cell lines. The addition of crosslinking resulted in diminished TOP2A signal from the resistant sample. (**g**) TOP2A DNA decatentation activity is increased in nuclear extracts from the drug-resistant cell line (log_2_(R/S)=1.3). Error bars represent s.d. (0.33) from six replicate reactions from three independent nuclear extract preparations. (**h**) Western blot analysis of TOP2A from nuclear extracts from drug-sensitive (S) and -resistant (R) cell lines. Full images of the blots (**a**,**f**,**h**) and gel (**g**) are shown in Supplementary Fig. 9.

Cancer cell lines resistant to TOP1 inhibitors often display decreased levels of TOP1 and corresponding increased activity and/or levels of topoisomerase 2 (TOP2A) accompanied by sensitivity to TOP2A inihibitors^10^,^42^. However, HeLa/SN100 cells displayed increased relative resistance to TOP2A inhibitors including etoposide, doxorubicin and mitoxantrone^3^. SILAC measurements revealed that expression levels of TOP1 and TOP2A did not significantly change between the chemoresistant and sensitive cell lines with log_2_(R/S) ratios of 0.31 and 0.29, respectively, agreeing with previous mRNA level measurments^3^. Quantitative western blot measurements of TOP2A provided a log_2_(R/S) of 0.26, in excellent agreement with global SILAC measurements (Fig. 5a). However, quantitative crosslinking measurements identified an intra-molecular crosslink in TOP2A between K489 and K798 with increased levels in the HeLa/SN100 cells with log_2_(R/S)=1.84 (Fig. 5b–d), indicating these sites were more frequently available for crosslinking in the chemoresistant cells. It is worth noting that this same crosslinked peptide pair from TOP2A was also identified in a previous study^21^. Interestingly, mapping these sites onto the crystal structure for TOP2A revealed that this crosslink spans the DNA-binding gate (Fig. 5e). Lysine 489, located in the TOPRIM domain of TOP2A, is directly involved in DNA binding, while K798 is located in the WHD domain on a flexible loop near the active site Y805. Interaction between these domains is necessary to form the competent DNA cleavage complex^43^. PTMs including phosphorylation, acetylation, ubiquitination and SUMOylation can alter the activity, stability and localization of TOP2A^44^. Occupancy of phosphorylation sites in the C-terminal domain of TOP2A have been shown to be critical in regulating the decatenating activity of TOP2A and have also been correlated with etoposide resistance^45^. Altered localization of TOP2A has been identified in cells resistant to the topoisomerase 2 targeting drugs etoposide and mitoxantrone^46^. Immunoprecipitation (IP) of TOP2A produced similar levels from sensitive and resistant cells, however adding crosslinking diminishes the signal from resistant cells (Fig. 5f). One possibility for this observed difference, supported in part by the quantitative crosslinking results, is that the crosslinking reaction is stabilizing different conformations of TOP2A in the sensitive and resistant cells which are not equally recognized by the anti-TOP2A antibody. As TOP2A activity is conformation dependent, an assay of the TOP2A activity in nuclear extracts prepared from the sensitive and chemoresistant cell lines was performed. TOP2A consistently displayed increased decatenation activity in nuclear extracts from the chemoresistant cells log_2_(R/S)=1.3 (Fig. 5g and Supplementary Fig. 5). Western blot analysis for TOP2A from the nuclear extracts indicated similar levels of TOP2A present in the extracts, but different band patterns were observed indicating a potential difference in proteolytic sensitivity of TOP2A (Fig. 5h). TOP2A can assist and/or substitute for TOP1 functions^47^, therefore the observed increase in TOP2A activity is likely a contributing factor in the mechanism of resistance in HeLa/SN100. Increased levels of the observed *in vivo* crosslinked TOP2A peptide pair correlated with increased activity, while neither the observed TOP2A protein abundance nor mRNA levels^3^ showed this correlation.

Crosslinked peptide pairs from additional complexes involved in DNA DSB repair were measured with increased levels in the chemoresistant cell line. This includes the facilitates chromatin transcription (FACT) complex, implicated to function in chromatin remodelling during transcription, DNA replication, recombination and repair^48^. The two comprising subunits of the FACT complex, Structure-Specific Recognition Protein 1 (SSRP1) and Suppressor of Ty (SPT16), were found crosslinked to each other with increased levels in the resistant cells (log_2_(R/S)=1.23). Global SILAC ratios for these proteins were log_2_(R/S)=0.64 and log_2_(R/S)=0.67, respectively. The FACT complex exhibits binding affinity and specificity to cisplatin-damaged DNA and modulates sensitivity of cells to cisplatin^49^. HeLa/SN100 cells were shown to have a twofold increased resistance to cisplatin compared to the parental HeLa cells^3^. SSRP1 has also been shown to play a functional role in the repair of DNA DSBs by homologous recombination^50^. Expression of the FACT complex has been associated with particularly aggressive cancers with poor prognosis and is the molecular target of the Curaxin class of anticancer compounds^51^. Quantitative crosslinking results suggest increased levels of FACT present in the resistant cells potentially plays a role in the MDR phenotype in HeLa/SN100.

### Quantitation of crosslinked peptides from IF proteins

**Figure 6:**
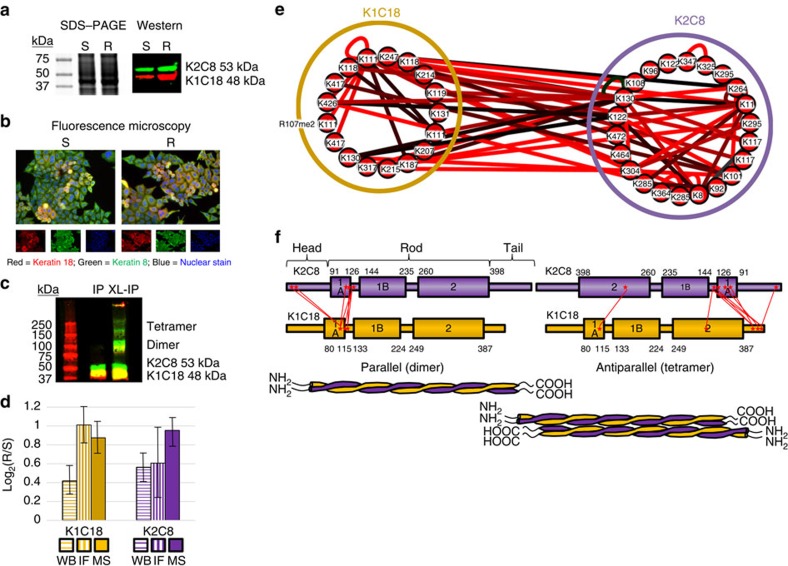
Analysis of intermediate filament proteins keratin 8/18. (**a**) Analysis of intermediate filament proteins keratins 8 and 18 (**a**) Coomassie stained one-dimensional SDS–PAGE and western blot probed with fluorescent anti-keratin 8 and anti-keratin 18 antibodies. (**b**) Composite widefield fluorescence microscope images of HeLa (S) and HeLa/SN100 (R). Scale bars, 20 μm. (**c**) Western blot analysis of co-immunoprecipitation of the keratin 8/18 complex with and without crosslinking (XL) from drug-sensitive cells. Adding crosslinking to the IP stabilized the heterodimer and tetramer complexes. (**d**) Bar chart comparing relative quantitative values obtained from western blotting (*n*=6), fluorescence microscopy (*n*=4) and global SILAC (*n*=6) values for keratin 8 and keratin 18. Error bars represent 95% CIs for the mean values. (**e**) Crosslinked peptide network displaying the intra-crosslinked and inter-crosslinked peptides identified for keratin 8 and keratin 18. Nodes and edges are coloured the same as in Fig. 2, with red indicating increased levels in the chemoresistant cell line. Crosslinked lysine residue numbers are indicated for each node. Loop edges indicate unambiguous homodimeric crosslinked peptides. (**f**) Mapping of inter-protein crosslinked sites onto the domain structures for keratin 8 and 18. 7 observed crosslinked peptides are supportive of a parallel orientation between keratin 8 and keratin 18 while 8 crosslinked peptides are supportive of an antiparallel orientation. Full images of the blot and gel (**a**) are shown in Supplementary Fig. 10.

Cytoskeletal proteins form the structural framework for cells, and provide a dynamic scaffold for mediating the localization and interactions of intracellular proteins. IF proteins expressed by epithelial cancer cells protect cells from mechanical stress as well as other cellular stressors that can lead to cell death including treatment with chemotherapeutic drugs thereby conferring an intrinsic chemoresistance^52^,^53^. Keratins are the primary IF component proteins in epithelial cells forming obligate heterodimer complexes between type 1 (acidic, keratins 9–28) and type 2 (basic, keratins 1–8 and 71–80) often displaying interdependent stability and expression^53^,^54^. In the current study we used global SILAC measurements, immunofluorescence microscopy and western blotting to confirm increased relative expression levels of both keratin 8 and 18 in HeLa/SN100 (Fig. 6a–d and Supplementary Fig. 6). Generally, the results from all three techniques are in agreement, with increased levels of keratins 8 and 18 in the chemoresistant cells.

IF complexes are highly dynamic, reorganizing during cellular events including differentiation, mitosis and apoptosis. Expression levels of keratins 8/18 have been used as diagnostic markers in cancer and prognostic indicators of cancer treatment, and several studies have implicated that keratins 8/18 play a role in MDR phenotypes in a variety of cancer cell types^55^,^56^,^57^,^58^. Fluorescence microscopy revealed that in addition to higher global levels of keratins 8/18 in the resistant cells, the cellular localization between the chemoresistant and sensitive cells was similar and the granular localization of these proteins characteristic of cells undergoing apoptosis^59^ was not observed (Fig. 6b). The addition of keratins 8/18 to cell lines lacking these cytoskeletal proteins induces MDR phenotypes and survival advantages against treatment with a variety of drugs including; mitoxantrone, doxorubicin, melphalan, bleomycin and mitomycin C^60^. Moreover, Cress and Dalton showed that cellular expression of keratin 8 or 18 alone (in the absence of an organized IF network) was enough to confer a MDR phenotype^52^. IP of keratin 8 also pulled down keratin 18 and combining crosslinking with the IP stabilized higher order complexes between the two proteins (Fig. 6c). The presence of keratins 8/18 in high mass bands from crosslinked IP samples was confirmed by LC–MS^2^ analysis of in-gel digests (Supplementary Fig. 7). MS analysis of enriched crosslinked peptide samples identified a total of 27 intermolecular crosslinked peptide pairs between keratins 8/18, several of which were significantly increased with MDR (Fig. 6e). Although much is known about the general domain structure and assembly of keratins 8/18, no detailed structures exist for these proteins. Intermediate filaments remain the least well studied and understood of the major cytoskeletal systems in terms of their structure and function^61^. IF proteins contain a central α-helical rod domain and non-α-helical N-terminal (head) and C-terminal (tail) domains connected by short disordered linker regions. The head and tail domains contain several PTM sites potentially important for signalling during the reorganization of IF structures. While phosphorylation of IF proteins has been the most well studied PTM to date, there is a growing appreciation of the diversity of PTMs and their role in the function, assembly and dynamics of the IF cytoskeleton^62^. During assembly of IF oligomers, keratins 8/18 arrange first into parallel heterodimers followed by antiparallel tetramers finally joining to form protofilaments^63^. Therefore, the intermolecular crosslinks between keratins 8/18 were segregated into parallel or antiparallel categories. Seven unique inter-protein crosslinks (site to site) were identified consistent with a parallel orientation of keratins 8/18, indicative of a heterodimer structure. Eight crosslinked sites were consistent with an antiparallel orientation supportive of a tetramer assembly (Fig. 6f). The remaining 12 crosslinked peptide pairs were ambiguous resulting from either parallel or antiparallel orientations. The log_2_(R/S) values for the crosslinked peptide pairs assigned to parallel and antiparallel orientations spanned similar ranges from 0.5 to 3.5, supporting an increased concentration of the assembled IF structure in the chemoresistant cells (Fig. 6f). Additionally, five unique unambiguous homodimeric crosslinked peptide pairs were identified in keratin 8 at residues K8, K11, K130, K285 and K325 all of which except K8 had increased levels in the resistant cell line. Only a single residue in keratin 18, K11, was identified in an unambiguous homodimeric crosslink. We identified three variations of the homodimer involving K11 including one with a dimethylation modification on R107 which was greatly increased in the chemoresistant cells with a log_2_(R/S) of 3.15 (Supplementary Fig. 8). Dimethylation of arginine residues has been previously identified on keratin 18 however not at R107 (ref. 62). This is evidence for a novel PTM on keratin 18 potentially playing a role in the dynamics of IF assembly in relation to chemoresistance, although follow up validation experiments will be needed to conclusively establish this. In addition to the keratin 8/18 interaction, several crosslinked peptides identify interactions on a wider IF protein network. For example keratins 8/18 were both crosslinked to keratin 7. Keratin 18 and keratin 7 were also identified crosslinked to vimentin and keratin 18 was crosslinked to plectin. Quantitative measurements on these additional IF protein crosslinked peptides did not appear to be increased with chemoresistance to the same extent as those between keratin 8 and 18 (Fig. 2 and Supplementary Data 1).

Although the exact molecular mechanism of resistance conferred by keratins 8/18 remains unknown, it is suspected to involve interference with apoptosis potentially through association with tumour necrosis factor and Fas receptor family members mediating apoptosis^59^. The keratins 8/18 IF network is rapidly reorganized during apoptosis by caspase-directed cleavage of keratin 18, resulting in the formation of keratin fragments that aggregate and are excreted from cells in the latter stages of apoptosis^64^. The immunochemical M30 and M65 assays, detect caspase cleaved keratin 18 fragments and have shown promise as clinically useful prognostic tools for treatment of various cancers^65^,^66^,^67^. The *in vivo* crosslinking results presented here suggest increased levels of the keratins 8/18 complex, accompanied by conformational changes potentially play a role in the MDR phenotype of HeLa/SN100.

## Discussion

The regulation of all crucial biological pathways is mediated by proteins. Changes to protein structures and PPIs are therefore key factors in the regulatory mechanism. To gain new insight into the regulation of pathways, the development of novel approaches that enable quantitation of PPIs and structural features as they exist inside cells is necessary. Here we have presented an *in vivo* quantitative crosslinking approach that provides these capabilities. The network shown in Fig. 2a is the first illustration of this capability. While this network is certainly not comprehensive in terms of all existing protein interactions, the detected crosslinked peptide pairs provide the first quantitative view on protein structural features and protein–protein interactions as they exist within cells. Knowledge of these molecular interactions along with the ability to observe changes in protein structures and interactions in drug resistant cancer cells yields many opportunities to improve understanding of *in vivo* function. When mapped onto existing protein structural models, identified crosslinked sites showed excellent agreement with known structures while those in proteins or regions of proteins with no existing structural data provide novel structural insights. Repeatable detection of this large number of crosslinked species in multiple biological replicates from resistant and sensitive cell lines demonstrates *in vivo* crosslinking experiments that can achieve high specificity and yield quantitative information on protein structures and complexes as they exist in cells. This capability can significantly advance molecular level understanding of phenotypes by enabling new information to be incorporated into edgotype characterization of interaction networks. Future studies employing this new methodology along with continuing technological advancements, will further advance knowledge toward a system-wide level and the ultimate goal of obtaining a molecular signature of drug resistance in cancer.

Inter-protein crosslinked peptides provide evidence for protein complexes for well-known interacting partners as well as new protein interactions. Quantitative measurements with SILAC and PIR technologies reveal changes to protein interaction topologies that occur with chemoresistance in human cancer cells that often correlate with global protein level. Such was the case with the IF proteins, keratins 8/18 that displayed parallel increases in intermolecular crosslinked peptide and protein abundance levels. Conversely, as shown for histone H3 and TOP2A, correlation between protein abundance and crosslinked peptide pair abundance were not observed, therefore regulatory mechanisms other than expression underpin these observed changes. In the case of TOP2A we have demonstrated that changes in crosslinked peptide pair levels can correlate with changes to enzymatic activity. Our results show that determination of regulatory changes at the level of PPIs in cells is possible with quantitative *in vivo* crosslink analysis. Furthermore, quantification of crosslinked peptides containing PTMs provides novel insight into PTM-induced changes in protein structure or localization, as anticipated from epigenetic regulatory mechanisms. Edgotype analysis by quantitative crosslinking and MS should be generalizable to other biological systems serving as a new tool to provide unique insight into the molecular analysis of biological phenotypes.

## Methods

### Crosslinking of drug-sensitive and -resistant cancer cells

Drug-sensitive (HeLa) and multidrug resistant (HeLa/SN100) cervical carcinoma cells^3^ were routinely cultured at 37 °C under a humidified atmosphere containing 5% CO_2_ in either isotopically light or heavy (Lys^13^C_6_^15^N_2_, Arg-^13^C_6_) SILAC DMEM containing 10% FBS and 1% penicillin/streptomycin. HeLa/SN100 cells were previously developed by continuous exposure to 100 nM SN-38 and their relative level of resistance measured for 16 different chemotherapeutic drugs^3^. The media for HeLa/SN100 was supplemented with 100 nM of SN-38. Cells were harvested at 80% confluency using 0.05% trypsin EDTA, were pelleted by centrifugation for 3 min at 300*g*, and the resulting cell pellets were washed three times with PBS (137 mM NaCl, 2.7 mM KCl, 10 mM Na_2_HPO_4_, 1.8 mM K_2_HPO_4_, pH=7.4). The PIR crosslinker, biotin aspartate proline n-hydroxyphthalimide (BDP-NHP), was synthesized as described in the Supplementary Methods^21^, and added to a 1:1 mixture of isotopically light and heavy cells at a final concentration of 10 mM. The crosslinking reaction was carried out for 1 h at room temperature with constant mixing. After crosslinking the cells were pelleted and washed three times with 100 mM NH_4_HCO_3_ to remove hydrolysed crosslinker. Samples were prepared in five biological replicates, including isotope label swapping.

### Crosslinked sample preparation and LC–MS analysis

To generate cell lysates, crosslinked cell samples were subjected to cryogenic grinding using a Retsch MM400 mixer mill using three 1 min cycles at 30 Hz. Proteins were extracted from the cryoground cell debris using an 8 M urea solution in 0.1 M Tris at pH 8.0. Sample viscosity was reduced by sonication using a GE-130 ultrasonic processor, followed by reduction and alkylation of cysteine residues by incubation with 5 mM *tris*(2-carboxyethyl)phosphine (TCEP) for 30 min followed by a 45 min incubation with 10 mM iodoacetamide. The urea concentration was lowered to <0.8 M by centrifugal filtration using Amicon Ultra filter units with a 30 kDa molecular weight cut off (Millipore, Billerica, MA, USA). Proteins were extracted from the filter with 0.1 M NH_4_HCO_3_ and digested with a 1:200 ratio of trypsin at 37 °C overnight. The peptide samples were then desalted using C18 Sep-Pak cartridges (Waters), followed by concentration and removal of acetonitrile by vacuum centrifugation using an EZ2-Plus evaporator (Genevac, Gardiner, NY, USA). The desalted peptide sample was fractionated by strong cation exchange chromatography using a 4.6 × 100 mM column packed with PolySULFOETHYL aspartamide (Nest Group, Southborough, MA, USA) coupled to an Agilent 1200 series HPLC. A flow rate of 1.5 ml min^−1^ was used to deliver a step gradient consisting of 5 min duration, 50 mM salt steps covering the concentration range from 0 to 500 mM of ammonium acetate containing 25% acetonitrile and 0.5% formic acid. Eluting strong cation exchange fractions were collected and acetonitrile was removed by vacuum centrifugation. The pH of the fractions was adjusted to 7.4 with the addition of 1 M NaOH. To each fraction, 300 μl of 50% UltraLink monomeric avidin slurry (Thermo/Pierce) was added and the sample was mixed at 800 r.p.m. for 30 min at room temperature. The immobilized avidin beads were washed ten times with 1 ml of 100 mM NH_4_HCO_3_ pH 8.0 followed by elution with 500 μl of 70% acetonitrile containing 0.5% formic acid. Enriched PIR-labelled peptides were then concentrated by vacuum centrifugation, resuspended in 50 μl of 0.1% formic acid and stored at −80 °C until LC–MS analysis.

PIR-crosslinked peptides were analysed in technical triplicate by LC–MS using a Waters NanoAcquity UPLC coupled to a Thermo Velos-FTICR mass spectrometer^68^ and a novel real-time adaptive, targeted mass spectrometry method developed for PIR-crosslinked peptides^19^. Briefly, peptides were loaded onto a 3 cm × 100 μm inner diameter fused silica trap column packed with a stationary phase consisting of Michrom Magic C8, 5 μm diameter, 200A pore size particles (Bruker) with a flow rate of 2 μl min^−1^ of mobile phase consisting of 98% solvent A (H_2_O containing 0.1% formic acid) and 2% solvent B (acetonitrile (ACN) containing 0.1% formic acid) for 10 min. Peptides were then fractionated over a 60 cm × 75 μm inner diameter fused silica analytical column packed with Michrom Magic C8, 5 μm diameter, 100A pore size particles by applying a linear gradient from 95% solvent A, 5% solvent B to 60% solvent A, 40% solvent B over either 120 or 240 min at a flow rate of 300 nl min^−1^. Eluting peptide ions were ionized by electrospray ionization by applying a positive 2 kV potential to a laser pulled spray tip at the end of the analytical column. The Velos-FTICR mass spectrometer was operated utilizing ReACT^19^ where ions with a charge state of four or greater were selected for high-resolution MS^2^ analysis in the ICR cell where an ‘on-the-fly' check of the observed fragment ion masses against the PIR mass relationship (mass precursor=mass reporter ion+mass peptide 1+mass peptide 2) is performed. Masses that satisfied the PIR relationship within a tolerance of 20 p.p.m. mass error triggered subsequent low resolution MS^3^ analyses of the released crosslinked peptide ions. For SILAC-based determination of protein levels, peptide samples from six biological replicates of non-crosslinked 1:1 mixtures of isotopically light and heavy HeLa and HeLa/SN100 cells were analysed in technical duplicate by LC–MS using a Waters NanoAcquity UPLC coupled to a Thermo LTQ-XL Orbitrap mass spectrometer as described in the Supplementary Information^69^.

### Crosslinked peptide data analysis and processing

Peptide fragmentation spectra generated through MS^3^ events in the ReACT analysis were searched against a subset of the UniProt reference proteome database (downloaded 05.11.12) for *Homo sapiens* containing both forward and reverse protein sequences (6,696 total sequences) using Sequest (UWPR2012.01). The 3,348 proteins included in the subset database were identified as putative PIR-reactive proteins as described in the Supplementary Methods and Supplementary Data 2)^21^. Sequest search parameters included; a 25 p.p.m. precursor mass tolerance allowing for the consideration of up to three ^13^C offsets, a 0.36 Da fragment ion mass tolerance, static modifications for the isotope-labelled amino acids Arg-^13^C_6_ (6.020129 Da) and Lys^13^C_6_^15^N_2_ (8.014199 Da) as well as carbamidomethylation of Cys (57.021464 Da), the variable modifications of oxidation (15.9949 Da) on Met, methylation of Lys and Arg (14.015650 Da), dimethylation of Lys (28.031300 Da), acetylation of Lys (42.010556 Da), trimethylation of Lys (42.046950 Da) and the BDP stump mass (197.0324 Da) on Lys and protein N termini, considering only fully tryptic peptide sequences and allowing for up to three missed cleavage sites. False discovery of crosslinked peptides was addressed by searching a concatenated database containing forward and reverse protein sequences as described in the Supplementary Methods^19^. Briefly, crosslinked peptide sequences were reported for cases where both peptide sequences contained an internal Lys residue modified by the crosslinker (197.0324 Da) and were assigned at <5% false discovery rate using a forward/reverse database search strategy. The global FDR was estimated to be ≤1% and was measured by allowing reverse peptide sequences that pass the 5% FDR threshold at the identification stage to be mapped to PIR relationships and taking the ratio of decoy crosslinked peptide pairs (either one or two reverse peptide sequences) to the total number of crosslinked peptide pairs.

Relative quantitative analysis of crosslinked peptide pairs between chemoresistant and sensitive conditions was performed using MassChroQ^70^. Accurate mass and retention time information for the light isotope forms of each crosslinked peptide pair identified was input. Isotope shifts included Arg-^13^C_6_ (6.020129 Da) and Lys^13^C_6_^15^N_2_ (8.014199 Da). Retention time alignment was performed across replicates using the Obiwarp method. Quantitation was performed using the area under the curve for extracted ion chromatograms for the MS^1^ signal from crosslinked peptide pair ions that were generated using a ppm tolerance of ±10 p.p.m. using the Zivy peak detection algorithm.

### Global SILAC data analysis

MS^2^ spectra from the global SILAC data collected on the LTQ-XL Orbitrap were searched against the full UniProt reference proteome database (downloaded 05.11.12) for *H. sapiens* containing both forward and reverse protein sequences (40,486 total sequences). Sequest search parameters included; a 25 p.p.m. precursor mass tolerance allowing for the consideration of up to three ^13^C offsets, a 1.0005 Da fragment ion mass tolerance, variable modifications for the isotope-labelled amino acids Arg-^13^C_6_ (6.020129 Da), Lys^13^C_6_^15^N_2_ (8.014199 Da) and oxidation (15.9949 Da) on Met. Static amino acid modifications included carbamidomethylation of Cys (57.021464 Da). Only fully tryptic peptide sequences were considered and allowing for up to two missed cleavage sites. Reported peptide matches were filtered at <1% FDR based on a target/decoy search strategy. Quantitation was performed with MassChroQ using the same parameters as described above for crosslinked peptides.

### Immunoprecipitation and western blotting

Cell lysates for immunoprecipitation were generated by lysing cells in IP lysis buffer containing 10 mM Tris, 1% triton-X-100, 1 mM EDTA, 100 mM NaCl and protease inhibitors (complete protease inhibitor cocktail, Roche). IP of keratin 8 was performed by incubating cell lysate from crosslinked and/or non-crosslinked cells (1.5 × 10^6^ cells per sample), with a 1:70 dilution of anti-cytokeratin 8 antibody (EP1628Y, Abcam) overnight at 4 °C with constant rotation. IP of TOP2A was performed with a 1:50 dilution of anti-TOP2A (4733, Cell Signaling Technology) overnight at 4 °C with constant rotation. Antibody–protein conjugates were pulled down by incubating samples with 10 μl of UltraLink Protein G resin (Thermo Scientific) for 2 h at room temperature. The Protein G resin was pelleted by centrifugation at 2,500*g* for 2.5 min and washed three times with 200 μl IP buffer. After washing, proteins were eluted by heating to 95 °C for 5 min in 30 μl Laemmli SDS–polyacrylamide gel electrophoresis (PAGE) sample buffer. Eluted proteins were then analysed by SDS–PAGE and western blotting as described below.

Protein samples (20 μg per well) were separated by one-dimensional SDS–PAGE using a Mini-PROTEAN TGX 4–20% precast gel (Bio-Rad) and applying 110 V constant for 1 h. Proteins were then either stained with Bio-Safe Coommassie or transferred by western blotting to an Immobilon-FL PVDF (polyvinylidene difluoride) membrane (Millipore) using a Trans-Blot SD semi-dry transfer cell operated at 150 mA constant current for 2 h. Western blot membranes were blocked for 1 h with PBS-T (PBS containing 0.1% Tween 20) containing 5% wt/vol BSA. Primary antibodies against Keratin 18 (DC10, Cell Signaling Technology), Keratin 8 (EP1628Y, Abcam) or Keratin 8 PA5-28985, Thermo Scientific) and TOP2A (D10G9, Cell Signaling Technology) were added at 1:2,000, 1:25,000 or 1:2,000 and 1:1,000 dilutions respectively and incubated with the membrane for 1 h at room temperature. The membrane was washed three times with PBS-T before adding secondary antibodies goat anti-rabbit IRDye 800CW and goat anti-mouse IRDye 680RD (LI-COR) at 1:10,000 dilutions. The membrane was then washed three times with PBS-T and once with PBS before analysis using a LI-COR Odyssey imaging system. Full images for all gels and blots are available in Supplementary Figs 9 and 10.

### Fluorescence microscopy

HeLa and HeLa/SN100 cells were grown on Matek 2.5 cm plates with 1.5 glass coverslips until 80% confluent. Growth media was aspirated off and the cells were washed with PBS. Cells were then fixed with 1 ml of 10% formalin for 20 min at room temperature. Fixed cells were washed three times with PBS and then blocked for 1 h in 1 ml of PBS containing 5% BSA and 0.3% triton X-100. Cells were then incubated with primary antibodies (1:250) anti-Keratin 8 and (1:800) anti-Keratin 18 overnight at 4 °C. Cells were washed three times with PBS before incubation with the secondary antibodies, Alexa Fluor 568 goat anti-rabbit and Alexa Fluor 488 goat anti-mouse (Life Technologies) at 1:1,000 dilutions for 1 h at room temperature. Cells were then washed three times with PBS before imaging using a Nikon TiE inverted widefield fluorescence microscope. Image analysis was performed using ImageJ.

### Topoisomerase II α activity assay

Nuclear extracts from 10^7^ drug-sensitive and -resistant HeLa cells were prepared (Supplementary Methods). The decatenation activity of TOP2A was measured using the TopoGEN TopoII assay kit (TopoGEN, Port Orange, FL). 300 ng of kinetoplast DNA was reacted varying amounts of nuclear extract (2–0.1 μl) in 20 μl reaction volumes for 30 min at 37 °C. The reaction was stopped by the addition of 4 μl stop buffer/gel loading dye (5% Sarkosyl, 0.125% bromophenol blue and 25% glycerol). Samples were then electrophoresed on a 1% agarose gel containing 0.5 μg ml^−1^ ethidium bromide. Gels were imaged using a MultiDoc-It M-26x system (UVP, Upland, CA, USA) and images were quantified using ImageJ (1.48v).

## Additional information

**Accession codes:** The mass spectrometry crosslinking data have been deposited to the ProteomeXchange Consortium via the PRIDE partner repository with the data set identifier PXD002378.

**How to cite this article:** Chavez, J. D. *et al*. Quantitative interactome analysis reveals a chemoresistant edgotype. *Nat. Commun*. 6:7928 doi: 10.1038/ncomms8928 (2015).

## Supplementary Material

Supplementary Figures, Supplementary Methods and Supplementary References Supplementary Figures 1-10, Supplementary Methods and Supplementary References

Supplementary Data 1 *in vivo* cross-linked peptide pairs identified in the present study.

Supplementary Data 2 Peptides used to identify PIR-reactive proteins (3348) that constitute a “stage 1 database” (3348 forward sequences plus 3348 reverse sequences = 6696 total) that was used to identify cross-linked peptides in Supplementary data 1.

## References

[b1] Holohan C., Van Schaeybroeck S., Longley D. B. & Johnston P. G. Cancer drug resistance: an evolving paradigm. Nat. Rev. Cancer 13, 714–726 (2013). 24060863 10.1038/nrc3599

[b2] Gillet J. P. & Gottesman M. M. Mechanisms of multidrug resistance in cancer. Methods Mol. Biol. 596, 47–76 (2010). 19949920 10.1007/978-1-60761-416-6_4

[b3] Takara K. . Molecular changes to HeLa cells on continuous exposure to SN-38, an active metabolite of irinotecan hydrochloride. Cancer Lett. 278, 88–96 (2009). 19201079 10.1016/j.canlet.2008.12.033

[b4] Pommier Y. Topoisomerase I inhibitors: camptothecins and beyond. Nat. Rev. Cancer 6, 789–802 (2006). 16990856 10.1038/nrc1977

[b5] Norris M. D. . Expression of multidrug transporter MRP4/ABCC4 is a marker of poor prognosis in neuroblastoma and confers resistance to irinotecan in vitro. Mol. Cancer Ther. 4, 547–553 (2005). 15827327 10.1158/1535-7163.MCT-04-0161

[b6] Maliepaard M. . Circumvention of breast cancer resistance protein (BCRP)-mediated resistance to camptothecins in vitro using non-substrate drugs or the BCRP inhibitor GF120918. Clin. Cancer Res. 7, 935–941 (2001). 11309344

[b7] Sugimoto Y., Tsukahara S., Oh-hara T., Isoe T. & Tsuruo T. Decreased expression of DNA topoisomerase I in camptothecin-resistant tumor cell lines as determined by a monoclonal antibody. Cancer Res. 50, 6925–6930 (1990). 2170010

[b8] Tsurutani J. . Point mutations in the topoisomerase I gene in patients with non-small cell lung cancer treated with irinotecan. Lung Cancer 35, 299–304 (2002). 11844605 10.1016/s0169-5002(01)00425-1

[b9] Chrencik J. E. . Mechanisms of camptothecin resistance by human topoisomerase I mutations. J. Mol. Biol. 339, 773–784 (2004). 15165849 10.1016/j.jmb.2004.03.077

[b10] Xu Y. & Villalona-Calero M. A. Irinotecan: mechanisms of tumor resistance and novel strategies for modulating its activity. Ann. Oncol. 13, 1841–1851 (2002). 12453851 10.1093/annonc/mdf337

[b11] West K. A., Castillo S. S. & Dennis P. A. Activation of the PI3K/Akt pathway and chemotherapeutic resistance. Drug Resist. Updat. 5, 234–248 (2002). 12531180 10.1016/s1368-7646(02)00120-6

[b12] Venkatesan K. . An empirical framework for binary interactome mapping. Nat. Methods 6, 83–90 (2009). 19060904 10.1038/nmeth.1280 PMC2872561

[b13] Vidal M., Cusick M. E. & Barabasi A. L. Interactome networks and human disease. Cell 144, 986–998 (2011). 21414488 10.1016/j.cell.2011.02.016 PMC3102045

[b14] Sahni N. . Edgotype: a fundamental link between genotype and phenotype. Curr. Opin. Genet. Dev. 23, 649–657 (2013). 24287335 10.1016/j.gde.2013.11.002 PMC3902775

[b15] Sinz A. Chemical cross-linking and mass spectrometry to map three-dimensional protein structures and protein-protein interactions. Mass Spectrom. Rev. 25, 663–682 (2006). 16477643 10.1002/mas.20082

[b16] Walzthoeni T., Leitner A., Stengel F. & Aebersold R. Mass spectrometry supported determination of protein complex structure. Curr. Opin. Struct. Biol. 23, 252–260 (2013). 23522702 10.1016/j.sbi.2013.02.008

[b17] Bruce J. E. In vivo protein complex topologies: sights through a cross-linking lens. Proteomics 12, 1565–1575 (2012). 22610688 10.1002/pmic.201100516 PMC3654877

[b18] Chavez J. D. . Cross-linking measurements of the Potato leafroll virus reveal protein interaction topologies required for virion stability, aphid transmission, and virus-plant interactions. J. Proteome Res. 11, 2968–2981 (2012). 22390342 10.1021/pr300041t PMC3402239

[b19] Weisbrod C. R. . In vivo protein interaction network identified with a novel real-time cross-linked peptide identification strategy. J. Proteome Res. 12, 1569–1579 (2013). 23413883 10.1021/pr3011638 PMC3925062

[b20] Zheng C. . Cross-linking measurements of in vivo protein complex topologies. Mol. Cell. Proteomics 10, M110 006841 (2011). 10.1074/mcp.M110.006841 PMC3205858 21697552

[b21] Chavez J. D., Weisbrod C. R., Zheng C., Eng J. K. & Bruce J. E. Protein interactions, post-translational modifications and topologies in human cells. Mol. Cell. Proteomics 12, 1451–1467 (2013). 23354917 10.1074/mcp.M112.024497 PMC3650351

[b22] Chavez J. D., Liu N. L. & Bruce J. E. Quantification of protein-protein interactions with chemical cross-linking and mass spectrometry. J. Proteome Res. 10, 1528–1537 (2011). 21222489 10.1021/pr100898e PMC3086679

[b23] Schmidt C. & Robinson C. V. A comparative cross-linking strategy to probe conformational changes in protein complexes. Nat. Protoc. 9, 2224–2236 (2014). 25144272 10.1038/nprot.2014.144 PMC4172966

[b24] Tang X. & Bruce J. E. A new cross-linking strategy: protein interaction reporter (PIR) technology for protein-protein interaction studies. Mol. Biosyst. 6, 939–947 (2010). 20485738 10.1039/b920876c PMC3075923

[b25] Tang X., Munske G. R., Siems W. F. & Bruce J. E. Mass spectrometry identifiable cross-linking strategy for studying protein-protein interactions. Anal. Chem. 77, 311–318 (2005). 15623310 10.1021/ac0488762

[b26] Ong S. E. . Stable isotope labeling by amino acids in cell culture, SILAC, as a simple and accurate approach to expression proteomics. Mol. Cell. Proteomics 1, 376–386 (2002). 12118079 10.1074/mcp.m200025-mcp200

[b27] Navare A. T. . Probing the protein interaction network of *Pseudomonas aeruginosa* cells by chemical cross-linking mass spectrometry. Structure 23, 762–773 (2015). 25800553 10.1016/j.str.2015.01.022 PMC4756656

[b28] Kaake R. M. . A new *in vivo* cross-linking mass spectrometry platform to define protein-protein interactions in living cells. Mol. Cell. Proteomics 13, 3533–3543 (2014). 25253489 10.1074/mcp.M114.042630 PMC4256503

[b29] Zheng C. . XLink-DB: database and software tools for storing and visualizing protein interaction topology data. J. Proteome Res. 12, 1989–1995 (2013). 23413830 10.1021/pr301162j PMC3744611

[b30] Pommier Y., Pourquier P., Urasaki Y., Wu J. & Laco G. S. Topoisomerase I inhibitors: selectivity and cellular resistance. Drug Resist. Updat. 2, 307–318 (1999). 11504505 10.1054/drup.1999.0102

[b31] Pandita T. K. & Richardson C. Chromatin remodeling finds its place in the DNA double-strand break response. Nucleic Acids Res. 37, 1363–1377 (2009). 19139074 10.1093/nar/gkn1071 PMC2655678

[b32] Wilting R. H. & Dannenberg J. H. Epigenetic mechanisms in tumorigenesis, tumor cell heterogeneity and drug resistance. Drug Resist. Updat. 15, 21–38 (2012). 22356866 10.1016/j.drup.2012.01.008

[b33] Esteller M. Cancer epigenomics: DNA methylomes and histone-modification maps. Nat. Rev. Genet. 8, 286–298 (2007). 17339880 10.1038/nrg2005

[b34] Jones C. & Baker S. J. Unique genetic and epigenetic mechanisms driving paediatric diffuse high-grade glioma. Nat. Rev. Cancer 14, 651–661 (2014). 10.1038/nrc3811 PMC4747023 25230881

[b35] Chen M. C. . The HDAC inhibitor, MPT0E028, enhances erlotinib-induced cell death in EGFR-TKI-resistant NSCLC cells. Cell Death Dis. 4, e810 (2013). 24052078 10.1038/cddis.2013.330 PMC3789188

[b36] Maiso P. . The histone deacetylase inhibitor LBH589 is a potent antimyeloma agent that overcomes drug resistance. Cancer Res. 66, 5781–5789 (2006). 16740717 10.1158/0008-5472.CAN-05-4186

[b37] Lane A. A. & Chabner B. A. Histone deacetylase inhibitors in cancer therapy. J. Clin. Oncol. 27, 5459–5468 (2009). 19826124 10.1200/JCO.2009.22.1291

[b38] Turner B. M. Reading signals on the nucleosome with a new nomenclature for modified histones. Nat. Struct. Mol. Biol. 12, 110–112 (2005). 15702071 10.1038/nsmb0205-110

[b39] Wang Z. . Combinatorial patterns of histone acetylations and methylations in the human genome. Nat. Genet. 40, 897–903 (2008). 18552846 10.1038/ng.154 PMC2769248

[b40] Fnu S. . Methylation of histone H3 lysine 36 enhances DNA repair by nonhomologous end-joining. Proc. Natl Acad. Sci. USA 108, 540–545 (2011). 21187428 10.1073/pnas.1013571108 PMC3021059

[b41] Pai C. C. . A histone H3K36 chromatin switch coordinates DNA double-strand break repair pathway choice. Nat. Commun. 5, 4091 (2014). 24909977 10.1038/ncomms5091 PMC4535359

[b42] Sugimoto Y., Tsukahara S., Oh-hara T., Liu L. F. & Tsuruo T. Elevated expression of DNA topoisomerase II in camptothecin-resistant human tumor cell lines. Cancer Res. 50, 7962–7965 (1990). 2174738

[b43] Dong K. C. & Berger J. M. Structural basis for gate-DNA recognition and bending by type IIA topoisomerases. Nature 450, 1201–1205 (2007). 18097402 10.1038/nature06396

[b44] Chen T., Sun Y., Ji P., Kopetz S. & Zhang W. Topoisomerase IIalpha in chromosome instability and personalized cancer therapy. Oncogene doi:10.1038/onc.2014.332 (2014). 10.1038/onc.2014.332 PMC4404185 25328138

[b45] Chikamori K. . Phosphorylation of serine 1106 in the catalytic domain of topoisomerase II alpha regulates enzymatic activity and drug sensitivity. J. Biol. Chem. 278, 12696–12702 (2003). 12569090 10.1074/jbc.M300837200

[b46] Rasheed Z. A. & Rubin E. H. Mechanisms of resistance to topoisomerase I-targeting drugs. Oncogene 22, 7296–7304 (2003). 14576839 10.1038/sj.onc.1206935

[b47] Vos S. M., Tretter E. M., Schmidt B. H. & Berger J. M. All tangled up: how cells direct, manage and exploit topoisomerase function. Nat. Rev. Mol. Cell Biol. 12, 827–841 (2011). 22108601 10.1038/nrm3228 PMC4351964

[b48] Garcia H. . Facilitates chromatin transcription complex is an ‘accelerator' of tumor transformation and potential marker and target of aggressive cancers. Cell Rep. 4, 159–173 (2013). 23831030 10.1016/j.celrep.2013.06.013 PMC5886782

[b49] Yarnell A. T., Oh S., Reinberg D. & Lippard S. J. Interaction of FACT, SSRP1, and the high mobility group (HMG) domain of SSRP1 with DNA damaged by the anticancer drug cisplatin. J. Biol. Chem. 276, 25736–25741 (2001). 11344167 10.1074/jbc.M101208200

[b50] Kumari A., Mazina O. M., Shinde U., Mazin A. V. & Lu H. A role for SSRP1 in recombination-mediated DNA damage response. J. Cell Biochem. 108, 508–518 (2009). 19639603 10.1002/jcb.22280 PMC8215854

[b51] Gasparian A. V. . Curaxins: anticancer compounds that simultaneously suppress NF-kappaB and activate p53 by targeting FACT. Sci. Transl. Med. 3, 95ra74 (2011). 10.1126/scitranslmed.3002530 PMC6281439 21832239

[b52] Cress A. E. & Dalton W. S. Multiple drug resistance and intermediate filaments. Cancer Metastasis Rev. 15, 499–506 (1996). 9034606 10.1007/BF00054015

[b53] Karantza V. Keratins in health and cancer: more than mere epithelial cell markers. Oncogene 30, 127–138 (2011). 20890307 10.1038/onc.2010.456 PMC3155291

[b54] Schweizer J. . New consensus nomenclature for mammalian keratins. J. Cell Biol. 174, 169–174 (2006). 16831889 10.1083/jcb.200603161 PMC2064177

[b55] Liu F. . Co-expression of cytokeratin 8 and breast cancer resistant protein indicates a multifactorial drug-resistant phenotype in human breast cancer cell line. Life Sci. 83, 496–501 (2008). 18725232 10.1016/j.lfs.2008.07.017

[b56] Hammer E. . Proteomic analysis of doxorubicin-induced changes in the proteome of HepG2cells combining 2-D DIGE and LC-MS/MS approaches. Proteomics 10, 99–114 (2010). 20017144 10.1002/pmic.200800626

[b57] Bichat F., Mouawad R., Solis-Recendez G., Khayat D. & Bastian G. Cytoskeleton alteration in MCF7R cells, a multidrug resistant human breast cancer cell line. Anticancer Res. 17, 3393–3401 (1997). 9413178

[b58] Bauman P. A., Dalton W. S., Anderson J. M. & Cress A. E. Expression of cytokeratin confers multiple drug resistance. Proc. Natl Acad. Sci. USA 91, 5311–5314 (1994). 7515497 10.1073/pnas.91.12.5311 PMC43984

[b59] Oshima R. G. Apoptosis and keratin intermediate filaments. Cell Death Differ. 9, 486–492 (2002). 11973607 10.1038/sj.cdd.4400988

[b60] Anderson J. M. . Cytokeratin expression results in a drug-resistant phenotype to six different chemotherapeutic agents. Clin. Cancer Res. 2, 97–105 (1996). 9816096

[b61] Goldman R. D., Cleland M. M., Murthy S. N., Mahammad S. & Kuczmarski E. R. Inroads into the structure and function of intermediate filament networks. J. Struct. Biol. 177, 14–23 (2012). 22120848 10.1016/j.jsb.2011.11.017 PMC3269975

[b62] Snider N. T. & Omary M. B. Post-translational modifications of intermediate filament proteins: mechanisms and functions. Nat. Rev. Mol. Cell Biol. 15, 163–177 (2014). 24556839 10.1038/nrm3753 PMC4079540

[b63] Parry D. A., Strelkov S. V., Burkhard P., Aebi U. & Herrmann H. Towards a molecular description of intermediate filament structure and assembly. Exp. Cell Res. 313, 2204–2216 (2007). 17521629 10.1016/j.yexcr.2007.04.009

[b64] Caulin C., Salvesen G. S. & Oshima R. G. Caspase cleavage of keratin 18 and reorganization of intermediate filaments during epithelial cell apoptosis. J. Cell Biol. 138, 1379–1394 (1997). 9298992 10.1083/jcb.138.6.1379 PMC2132555

[b65] de Haas E. C. . Clinical evaluation of M30 and M65 ELISA cell death assays as circulating biomarkers in a drug-sensitive tumor, testicular cancer. Neoplasia 10, 1041–1048 (2008). 18813353 10.1593/neo.08620 PMC2546590

[b66] Stoetzer O. J. . Prediction of response to neoadjuvant chemotherapy in breast cancer patients by circulating apoptotic biomarkers nucleosomes, DNAse, cytokeratin-18 fragments and survivin. Cancer Lett. 336, 140–148 (2013). 23612068 10.1016/j.canlet.2013.04.013

[b67] Olofsson M. H. . Cytokeratin-18 is a useful serum biomarker for early determination of response of breast carcinomas to chemotherapy. Clin. Cancer Res. 13, 3198–3206 (2007). 17545523 10.1158/1078-0432.CCR-07-0009

[b68] Weisbrod C. R. . Performance evaluation of a dual linear ion trap-Fourier transform ion cyclotron resonance mass spectrometer for proteomics research. J. Proteomics 88, 109–119 (2013). 23590889 10.1016/j.jprot.2013.04.009 PMC3972134

[b69] Chavez J. D., Hoopmann M. R., Weisbrod C. R., Takara K. & Bruce J. E. Quantitative proteomic and interaction network analysis of cisplatin resistance in HeLa cells. PLoS One 6, e19892 (2011). 21637840 10.1371/journal.pone.0019892 PMC3102677

[b70] Valot B., Langella O., Nano E. & Zivy M. MassChroQ: a versatile tool for mass spectrometry quantification. Proteomics 11, 3572–3577 (2011). 21751374 10.1002/pmic.201100120

