# Supplementary material for: Quantitative interactome analysis reveals a chemoresistant edgotype
Source: Nat Commun. 2015 Aug 3;6:7928. doi: 10.1038/ncomms8928 (PMC4532879; doi:10.1038/ncomms8928)
Supplement: Supplementary Figures, Supplementary Methods and Supplementary References — Supplementary Figures 1-10, Supplementary Methods and Supplementary References [file ncomms8928-s1.pdf]

Supplementary Information

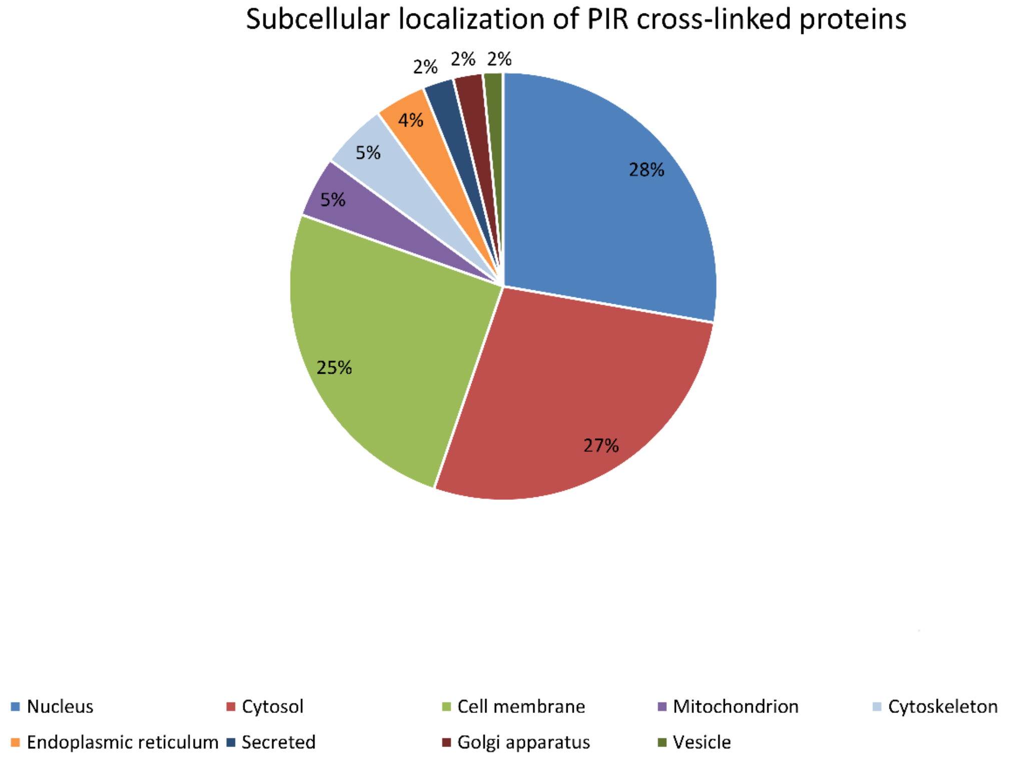

Supplementary Figure 1 | Subcellular localization of PIR cross-linked proteins. Pie chart indicating subcellular localization (derived from UniProt annotation) of the cross-linked proteins identified in this study. Consistent with our previous study[1] the majority of cross-links identified were from nuclear proteins followed by cytosolic and membrane proteins.

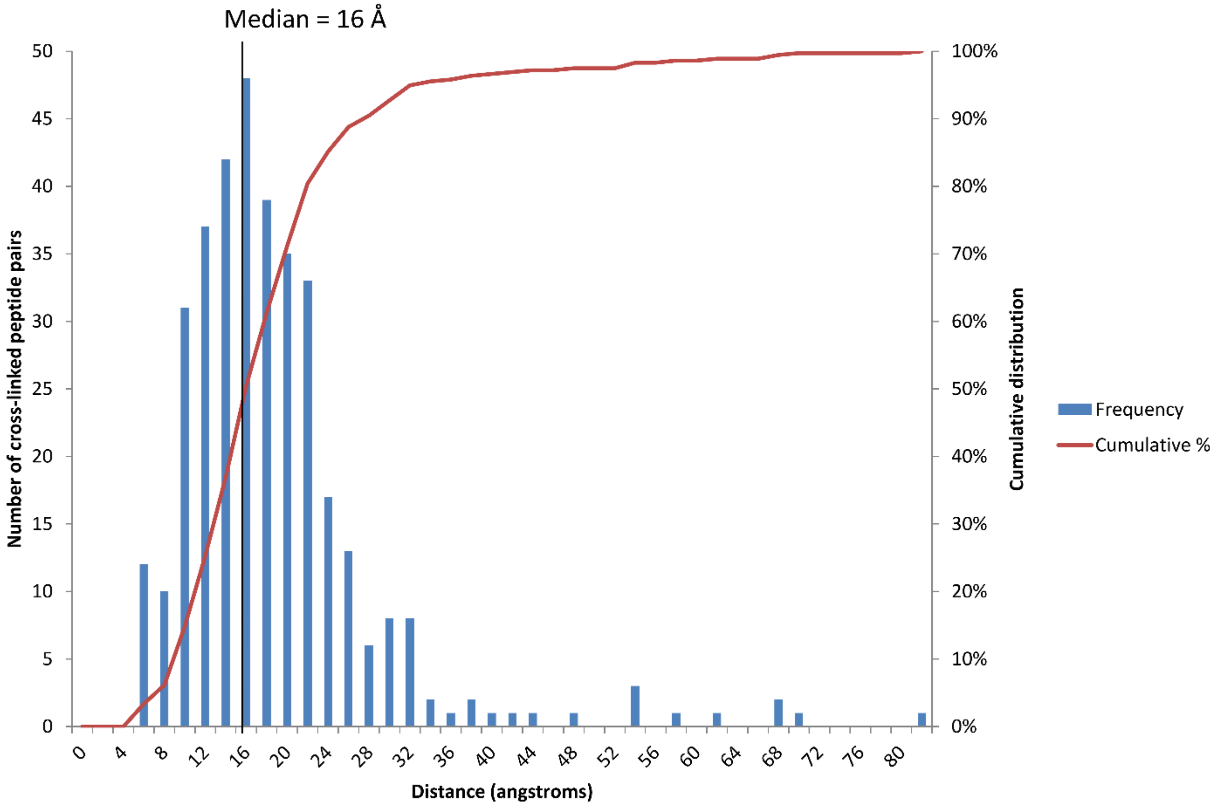

Supplementary Figure 2 | Euclidean Cα-Cα Distances for cross-linked sites. Distribution of distances for 357 unique cross-linked peptide pairs that were mapped to existing structures in the PDB using XlinkDB[2]. The median distance is 16 angstroms with 95% of the links being less than the linker arm distance of 35 angstroms. This is in agreement with our previous published results[1-3].

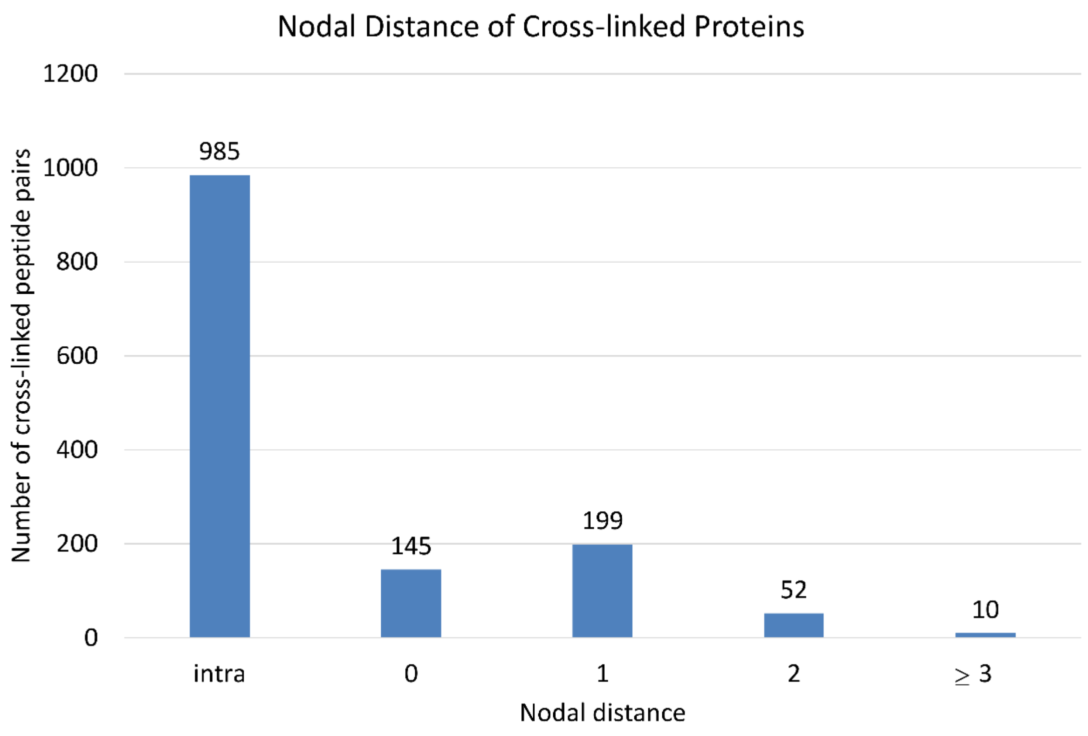

Supplementary Figure 3 | Nodal distance of cross-linked peptide pairs. Distribution of nodal distance comparing 1391 cross-links identified in this study to known interactions from the following databases; MIPS, DIP, IntAct, MINT, HPRD, or BioGRID. Intra-protein linkages are the most common as to be expected as the cross-linker has a higher probability to react with a nearby lysine side chain within the same protein molecule. For inter-protein cross-links a nodal distance of 0 represents an interaction from our data set that is also present in one of the databases mentioned above. Nodal distances of 1 or more represent potential novel interactions discovered through our study.

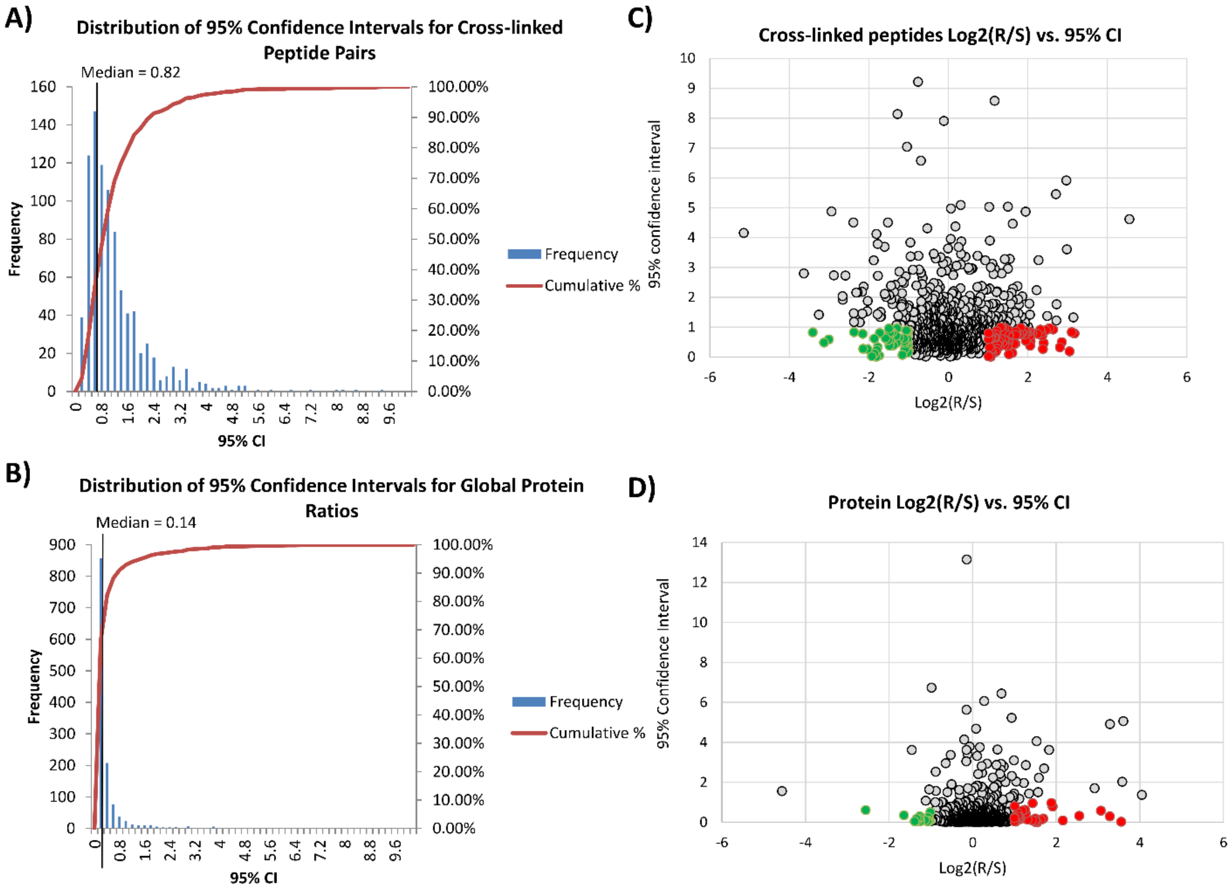

Supplementary Figure 4 | Variability of quantified cross-linked peptide pairs. Distribution of 95% confidence intervals for the Log_2(Resistant/Sensitive) (R/S) SILAC ratios obtained for **A)** cross-linked peptide pairs and **B)** global protein levels. **C)** Scatter plot of the Log2(R/S) values for cross-linked peptide pairs vs. the 95% confidence intervals. Those with Log2(R/S) values greater than 1 with CI's less than 1 are colored red. Cross-linked peptide pairs with Log2(R/S) values less than -1 and CI's less than 1 are colored green. **D)** Scatter plot for protein Log2(R/S) obtained by traditional SILAC analysis vs. 95% confidence interval. Color scheme for **D** is the same as **C**.

A)

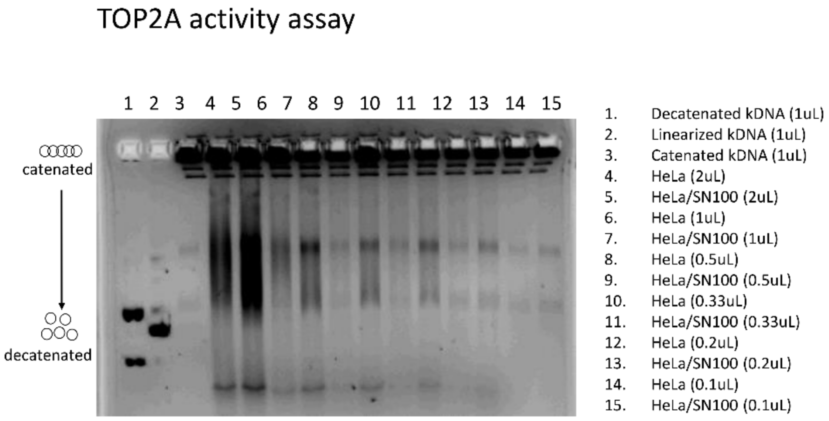

B)

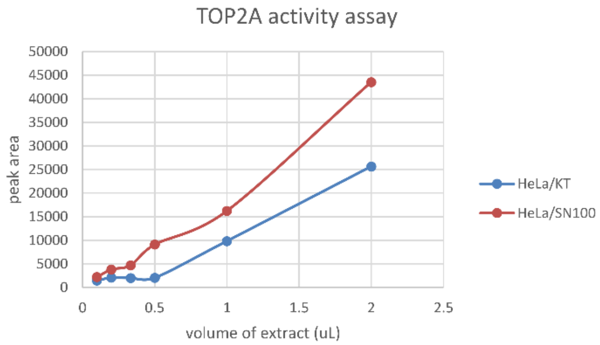

Supplementary Figure 5 | TOP2A activity assay. A) Image of an ethidium bromide stained gel from TOP2A decatenation activity assay. Varying amounts of nuclear extract from HeLa and HeLa/SN100 were incubated with catenated kDNA and the products separated on a 1% agarose gel. **B)** Quantitation of the decatenated kDNA products reveals that nuclear extracts from HeLa/SN100 contain increased TOP2A activity relative to nuclear extracts from HeLa.

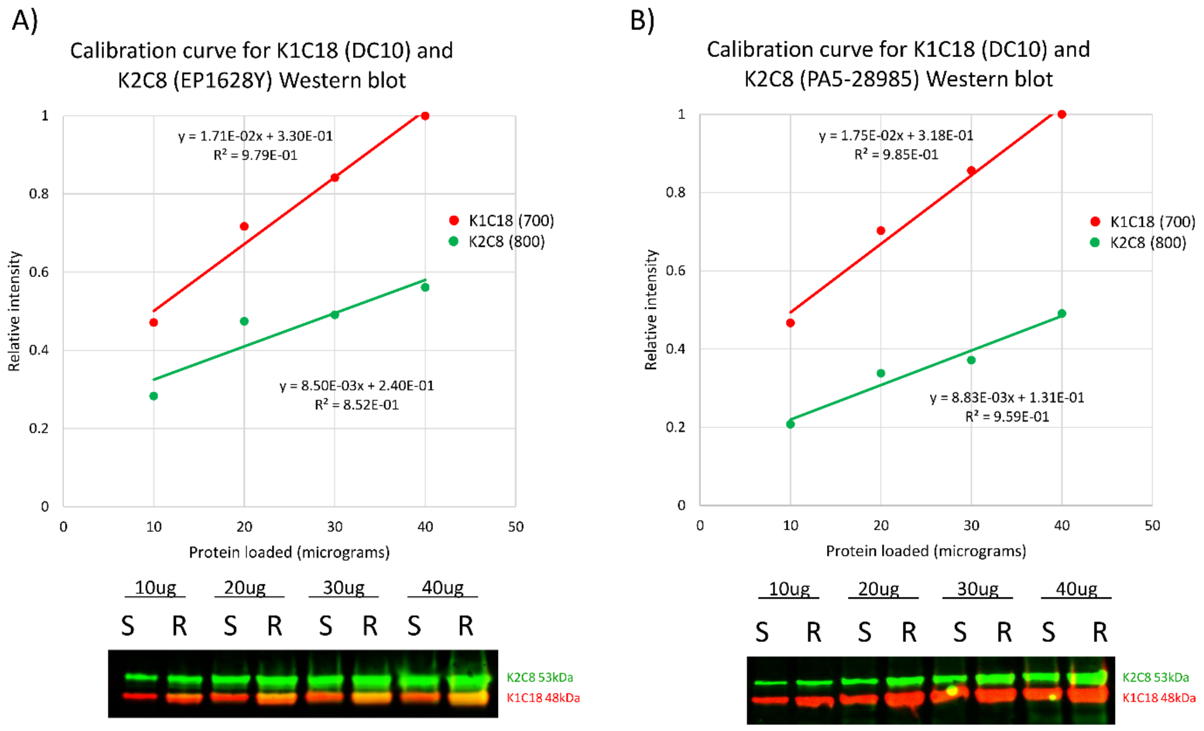

Supplementary Figure 6 | Keratin Western blot calibration curves. Calibration curves indicating a difference in sensitivity for the detection of keratin 8 and keratin 18 using two different primary antibody systems: **A)** anti-K1C18 (DC10) and **B)** anti-K2C8 (EP1628Y) and anti-K1C18 (DC10) and anti-K2C8 (PA5-28985). Full blot images are included in Supplementary Fig. 10.

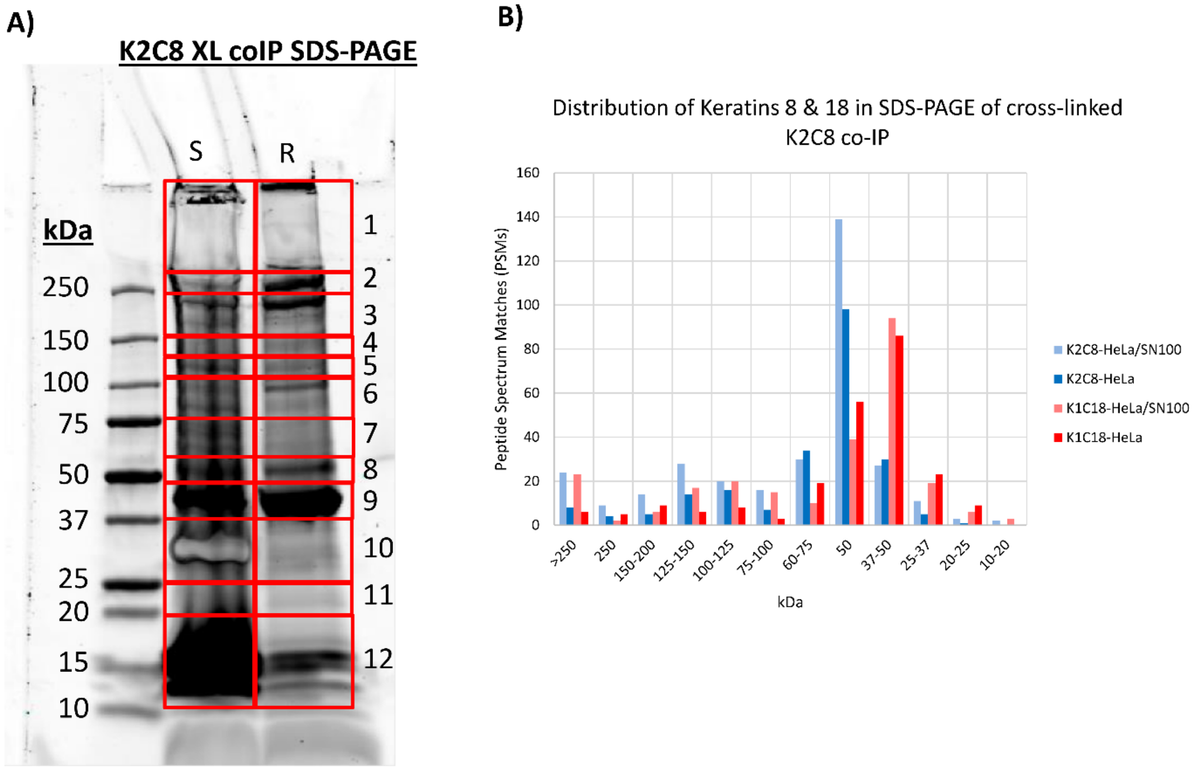

Supplementary Figure 7 | Cross-linking stabilizes higher order kertain 8/18 complexes. A) Commassie stained SDS-PAGE separation of PIR cross-linked K2C8 co-IP samples from both sensitive and resistant cells. Indicated sections were from the gel were excised, subjected to in-gel tryptic digestion and analyzed by LC-MS^2. **B)** Bar plot indicating the number of peptide spectrum matches for K2C8 and K1C18 identified from the various gel sections. The presence of both proteins in higher mass bands is supportive of the cross-linked stabilized dimer and tetramer forms of the K2C8 and K1C18 complex detected by Western blot in **Figure 6C**.

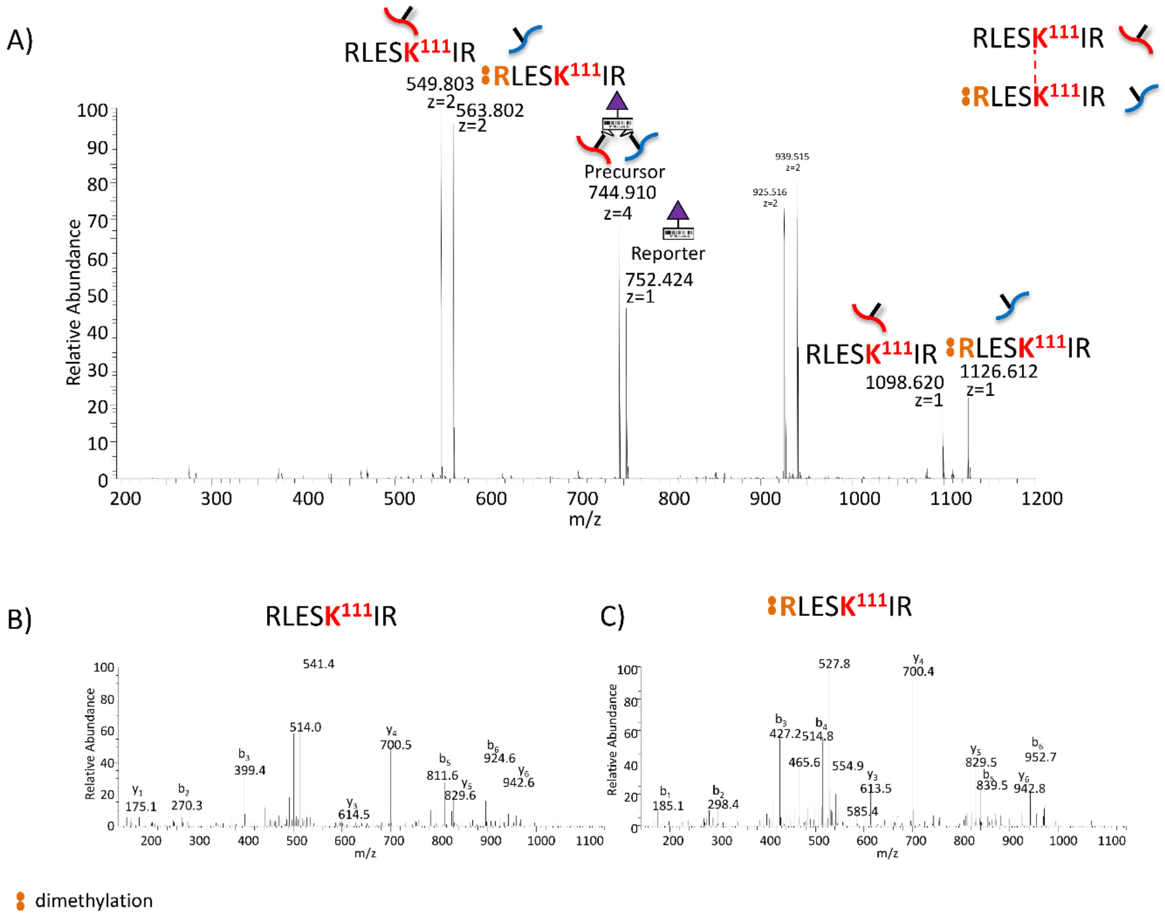

Supplementary Figure 8 | Identification of a homodimer cross-linked peptide pair from keratin 18 containing demethylation modification of R107. A) High resolution MS^2 spectrum for the unambiguous homodimeric cross-link involving K111 of keratin 18 where the peptide from one of the peptides also contains a dimethylation modification on R107. **B)** Low resolution MS^3 spectrum of the 549.8 m/z precursor ion, identifying the sequence RLESKIR spanning residues 107-113 of K2C8, and localizing the cross-linker reactive site to K111. **C)** Low resolution MS^3 spectrum of the 563.8 m/z precursor ion, identifying the sequence RLESKIR spanning residues 107-113 of K2C8 and localizing a dimethylation modification on R107 and the cross-linker reactive site on K111. The b ion series in this spectrum is shifted by 28 Da relative to those in "B)" localizing the PTM to the N-terminal arginine.

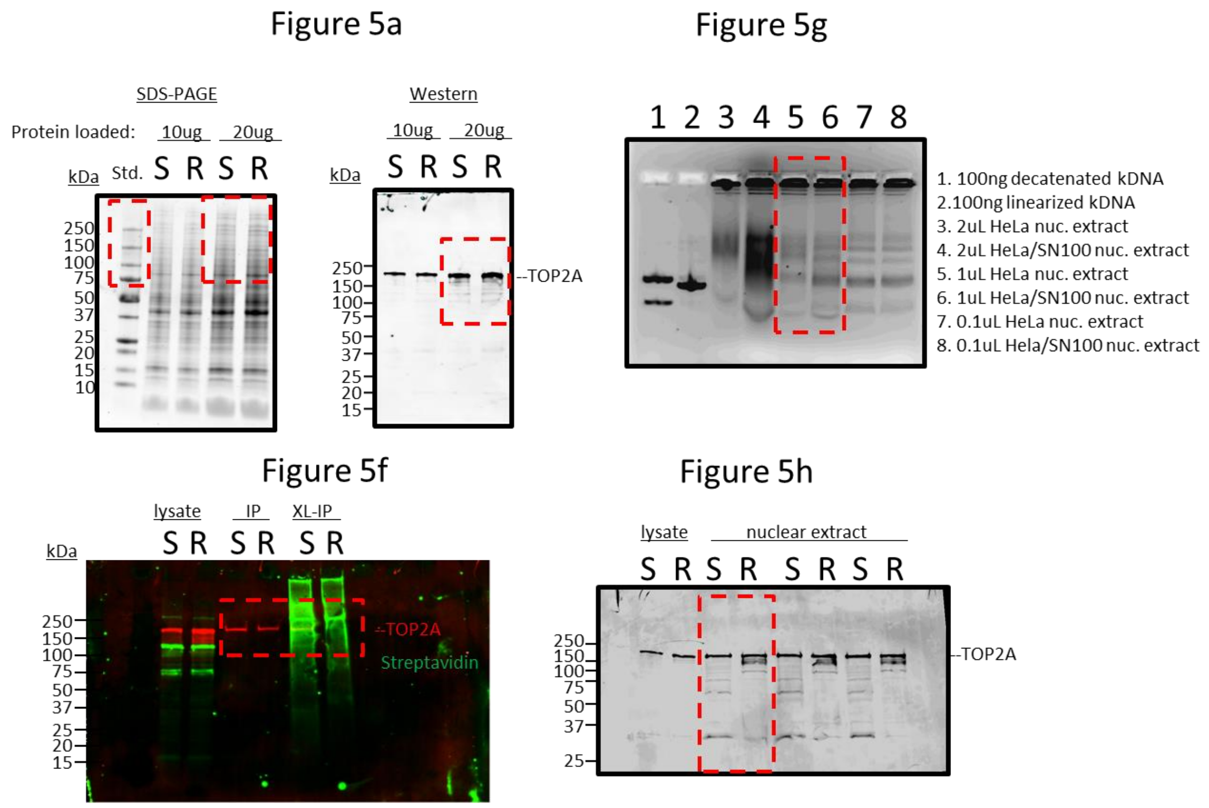

Supplementary Figure 9 | Representative original images for gel and blot analyses for Fig. 5

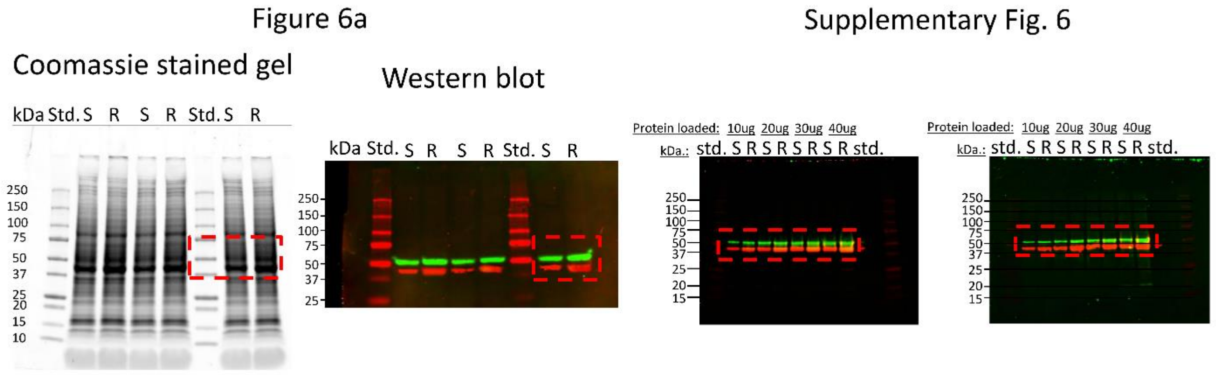

Supplementary Figure 10 | Representative original images for gel and blot analyses for Fig. 6 and Supplementary Fig. 6.

Supplementary Methods

Generation of a stage 1 database of putative PIR reactive proteins

100 µL of UltraLink monomeric avidin slurry was added to one milligram of cross-linked protein extracted from PIR cross-linked HeLa and/or HeLa/SN100 cells and incubated at room temperature for 30 min. The monomeric avidin beads were washed 5 times with 1mL of 0.1 M NH_4HCO_3 pH 8.0 before eluting PIR reactive proteins using 100uL of 8M urea in 0.1 M tris pH 8.0. Disulfide bonds in the eluted proteins were reduced with 5 mM TCEP for 30 minutes followed by alkylation with 10 mM iodoacetamide for 45 minutes. Samples were diluted 10x with 0.1 M NH_4HCO_3 pH 8.0 before overnight digestion with a 1:200 ratio of trypsin at 37°C. The peptide samples were then desalted using C18 Sep-Pak cartridges, followed by concentration and removal of acetonitrile by vacuum centrifugation using an EZ2-Plus evaporator.

Peptide samples were analyzed in triplicate using a Waters nanoAcquity UPLC coupled to a Velos-FTICR system[4]. Peptide samples were loaded onto a trap column (3 cm x 100 µm i.d.) packed with 200 Å Magic- C18AQ using a flow rate of 2 µl min^{-1} of 99% solvent A (H_2O containing 0.1% formic acid) and 1% solvent B (acetonitrile containing 0.1% formic acid) where they were washed for a total of 10 min. Peptides were then eluted from the trap column and separated by reversed-phase chromatography over a pulled tip, fused silica analytical column (60 cm x 75 µm i.d.) packed with 100 Å Magic- C18AQ at a flow rate of 300 nL min^{-1} using a linear gradient from 95% solvent A/5% solvent B to 60% solvent A/40% solvent B over 120 min. Data dependent analysis (DDA) with the Velos-FTICR mass spectrometer consisted of a high resolution (50,000 RP) MS^1 scan followed by low resolution MS^2 analysis on the 20 most intense precursors. MS^2 settings included a normalized collision energy of 35V, isolation width of 2, activation time of 10 ms, activation Q of 0.25 and a minimum signal threshold of 10,000. Charge state exclusion was applied for singly charged precursor ions and those with

undetermined charge state. Dynamic exclusion was enabled with settings including an exclusion window of 0.5 m/z low to 1.5 m/z high, exclusion time of 45 s, a list size of 500, and a repeat count of 1.

Resulting mass spectral data was searched against the full UniProt reference proteome database (downloaded 05.11.12) for *Homo sapiens* containing both forward and reverse protein sequences (40486 total sequences). Sequest search parameters included; a 25 ppm precursor mass tolerance allowing for the consideration of up to three ^{13}C offsets, a 1.0005 Da fragment ion mass tolerance, variable modifications oxidation (15.9949 Da) on Met. Static amino acid modifications included carbamidomethylation of Cys (57.021464 Da). Only fully tryptic peptide sequences were considered and allowing for up to 2 missed cleavage sites. Reported peptide matches were filtered at less than 1% FDR based on a target/decoy search strategy. This resulted in the identification of 3348 proteins that were used to construct the stage 1 database that was used for searching the MS^3 spectra generated from ReACT analysis of cross-linked peptides as described in the Methods of the main manuscript. Results from the stage 1 database analysis are summarized in **Supplementary Data 2**.

Estimation of false discovery rate for cross-linked peptide pairs

Sequest search results of the ReACT generated MS^3 spectra against the stage 1 database consisting of 3348 protein sequences (database contains both forward and reverse protein sequences for a total size of 6696 sequences) were filtered at the individual peptide level to less than 5% FDR. The individual peptide sequences were then mapped back to the PIR mass relationships (mass peptide 1 + mass peptide 2 + mass reporter) that were generated during LC-MS data acquisition using ReACT. Both forward and reverse peptide sequences passing the first 5% FDR threshold were allowed to map to cross-linked peptide mass relationships. The resulting cross-linked peptide pairs could then consist of two forward peptide sequences (fwd-fwd), a forward peptide sequence and reverse peptide sequence (fwd-rev) or two reverse peptide sequences (rev-rev), the first type considered as a target identification while the latter two considered as decoy identifications. The estimate for global FDR for the identification of cross-linked peptides was calculated as 100 * (decoy/(target+decoy)).

Synthesis of PIR cross-linker

The PIR cross-linker Biotin Aspartate Proline (BDP) synthesis was accomplished using solid phase peptide synthesis using an Endeavor 90 system (APPTEC, Louisville, KY) employing 9-fluorenylmethyloxycarbonyl (Fmoc) chemistry. The super acid sensitive SASRIN-glycine resin was used for the solid support. Amino acid additions to the resin occur in sequential order and are the following, Fmoc-Lys (Biotin), $Fmoc_2$-Lys, Fmoc-Pro, Fmoc-Asp (t-BOC), and succinic anhydride. Reaction yield at each coupling step was monitored via the absorbance of released FMOC at 307 nm with a cumulative measured yield of >90%. The activated n-hydroxyphthalamide (NHP) ester form of the cross-linker is synthesized in a final esterification step immediately prior to use with TFA-NHP. Cleavage of BDP from the solid support and removal of the *N-tert*-butoxycarbonyl (t-BOC) protecting groups from the Asp side chains was performed simultaneously using 95% trifluoroacetic acid 5% dichloromethane. Purification of BDP was performed via diethyl ether precipitation using 1:15 (cleavage mixture: diethyl ether). Diethyl ether solution was centrifuged at 3400 g to pellet precipitate. Diethyl ether was decanted and pellet was dried to yield BDP-NHP ester.

Global SILAC analysis on LTQ-Orbitrap

Peptide samples were loaded onto a trap column (3 cm × 75 µm i.d. packed with Michrom Magic C18AQ 200 Å pore size, 5 µm) and washed for 10 minutes at a flow rate of 2 µL min^{-1} with 98% solvent A (H_2O, 0.1% formic acid) and 2% solvent B (acetonitrile, 0.1% formic acid) using a nanoAcquity UPLC (Waters, Milford MA). Peptides were then fractionated over the analytical column (30 cm × 75 µm i.d. packed with Michrom Magic C18AQ 100 Å pore size, 5 µm particles) using a 120 minute linear gradient from 95% solvent A, 5% solvent B to 60% solvent A, 40% solvent B at a flow rate of 300 nL min^{-1}. Peptides were ionized by electrospray ionization (ESI) using a spray voltage of 2.0 kV. Data dependent

mass spectrometric analysis of SILAC samples was performed using a LTQ-Orbitrap mass spectrometer (Thermo). Full MS scans from 400–1400 *m/z* were performed in the Orbitrap mass analyzer with the resolution set to 60,000. Tandem mass spectrometry was performed in the ion trap mass analyzer on the five most abundant precursors detected in the Orbitrap full MS scan. Collision induced dissociation was performed using a normalized collision energy of 35 with a 30 ms activation time and an activation Q of 0.25. Ions selected for MS/MS sequencing were then dynamically excluded from repeated MS/MS events for 60 seconds, using an asymmetric mass window of 0.1 *m/z* on the low side and 1.1 *m/z* on the high side. All samples were analyzed in technical duplicate. Data was analyzed as described in the **Methods**.

Preparation of nuclear extracts

Nuclear extracts from 10^7 drug sensitive and resistant HeLa cells were prepared as follows. Cells were scraped into the medium and pelleted by centrifugation at 800 xg for 3 minutes at 4°C. Cell pellets were resuspended in 3 mL of ice cold TEMP buffer (10 mM Tris-HCl, pH 7.5, 1 mM EDTA, 4 mM $MgCl_2$, 0.5 mM PMSF) followed by centrifugation at 800 xg for 3 minutes at 4°C. The cell pellets were then resuspended in 3 mL cold TEMP buffer and incubated on ice for 10 minutes. Cell membranes were ruptured using a dounce homogenizer (8 strokes) and the presence of nuclei was confirmed by phase contrast microscopy. Nuclei were pelleted by centrifugation at 1500 xg for 10 minutes at 4°C. The nuclear pellet was resuspended in 1 mL of cold TEMP buffer and pelleted again at 1500 xg for 10 minutes at 4°C. The nuclear pellets were then suspended in 60 μL (3 x pellet volume) of ice cold TEP buffer (same as TEMP but lacking $MgCl_2$). 60 μL of ice cold 1 M NaCl was added, the sample was vortexed briefly and incubated on ice for 60 min. Samples were then centrifuged at 15,000 xg for 15 minutes at 4°C. The resulting supernatant was used for the TopoGEN TOP2A activity assay described in the **Methods**.

Supplementary References

1. Chavez, J. D., Weisbrod, C. R., Zheng, C., Eng, J. K. & Bruce, J. E. Protein interactions, post-translational modifications and topologies in human cells. *Molecular & cellular proteomics : MCP* **12**, 1451-67 (2013).
2. Zheng, C. et al. XLink-DB: Database and Software Tools for Storing and Visualizing Protein Interaction Topology Data. *Journal of proteome research* (2013).
3. Weisbrod, C. R. et al. In vivo protein interaction network identified with a novel real-time cross-linked peptide identification strategy. *J Proteome Res* **12**, 1569-79 (2013).
4. Weisbrod, C. R., Hoopmann, M. R., Senko, M. W. & Bruce, J. E. Performance evaluation of a dual linear ion trap-Fourier transform ion cyclotron resonance mass spectrometer for proteomics research. *J Proteomics* **88**, 109-19 (2013).
